# Area-Level Deprivation and Overall and Cause-Specific Mortality: 12 Years’ Observation on British Women and Systematic Review of Prospective Studies

**DOI:** 10.1371/journal.pone.0072656

**Published:** 2013-09-24

**Authors:** Maria T. Sánchez-Santos, Marco Mesa-Frias, Minkyoung Choi, Eveline Nüesch, Angel Asunsolo-Del Barco, Antoinette Amuzu, George Davey Smith, Shah Ebrahim, David Prieto-Merino, Juan P. Casas

**Affiliations:** 1 Department of Surgery, Medical and Social Sciences, Faculty of Medicine, University of Alcala, Madrid, Spain; 2 Oxford NIHR Musculoskeletal Biomedical Research Unit, Nuffield Department of Orthopaedics, Rheumatology and Musculoskeletal Sciences, University of Oxford, Headington, Oxford, United Kingdom; 3 Department of Non-communicable Disease Epidemiology, Faculty of Epidemiology and Population Health, London School of Hygiene and Tropical Medicine, London, United Kingdom; 4 Department of Social and Environmental Health Research, Faculty of Public Health and Policy, London School of Hygiene and Tropical Medicine, London, United Kingdom; 5 MRC Centre for Causal Analyses in Translational Epidemiology, School of Social and Community Medicine, University of Bristol, Bristol, United Kingdom; 6 South Asia Network for Chronic Disease, Public Health Foundation of India, New Delhi, India; 7 Institute of Cardiovascular Science, University College London, London, United Kingdom; Iran University of Medical Sciences, Iran (Islamic Republic Of)

## Abstract

**Background:**

Prospective studies have suggested a negative impact of area deprivation on overall mortality, but its effect on cause-specific mortality and the mechanisms that account for this association remain unclear. We investigate the association of area deprivation, using Index of Multiple deprivation (IMD), with overall and cause-specific mortality, contextualising findings within a systematic review.

**Methods And Findings:**

We used data from 4,286 women from the British Women’s Heart Health Study (BWHHS) recruited at 1999-2001 to examine the association of IMD with overall and cause-specific mortality using Cox regression models. One standard deviation (SD) increase in the IMD score had a hazard ratio (HR) of 1.21 (95% CI: 1.13-1.30) for overall mortality after adjustment for age and lifecourse individual deprivation, which was attenuated to 1.15 (95% CI: 1.04-1.26) after further inclusion of mediators (health behaviours, biological factors and use of statins and blood pressure-lowering medications). A more pronounced association was observed for respiratory disease and vascular deaths. The meta-analysis, based on 20 published studies plus the BWHHS (n=21), yielded a summary relative risk (RR) of 1.15 (95% CI: 1.11-1.19) for area deprivation (top [least deprived; reference] vs. bottom tertile) with overall mortality in an age and sex adjusted model, which reduced to 1.06 (95% CI: 1.04-1.08) in a fully adjusted model.

**Conclusions:**

Health behaviours mediate the association between area deprivation and cause-specific mortality. Efforts to modify health behaviours may be more successful if they are combined with measures that tackle area deprivation.

## Introduction

Health-related behaviours such as unhealthy diet, tobacco smoking, alcohol consumption and low physical activity are associated with major causes of avoidable mortality in middle-age and older people [1,2]. It is well established that individual deprivation (measured by individual socioeconomic position (SEP)) increases overall and cause-specific mortality, mainly explained through its effects on health-related behaviours [3].

More recently, the scope has been expanded to study how the socioeconomic environment of an area affects health of its residents, independent of deprivation at individual level [4,5]. Such studies have argued that the health of an individual in a specific area not only depends on individual characteristics but also on the deprivation in the area where the individual lives.

Many of the studies to date evaluating the association of area-level deprivation and health outcomes have adopted an ecological design. Although these studies have consistently found an association with overall and cause-specific mortality [6,7], these studies by the nature of their design were able neither to control for individual deprivation, nor to explore the impact that health-related behaviours have on the association of interest. Some prospective studies suggested the existence of a positive association between area-level deprivation and overall mortality [8,9]. However, not all the studies adjusted for individual deprivation, and those that adjusted often used incomplete measures of individual SEP [10,11]. Moreover, the association of area-level deprivation with cause-specific mortality has been infrequently examined and a greater uncertainty has been found in prospective studies that reported on it [12,13].

It is important to note that previous prospective studies have used different methods to measure deprivation at area-level, and many of those studies used readily available information related to area-level deprivation, rather than specific instruments designed for such purpose. The use of inconsistent and less reliable measures of area-level deprivation used in published studies may have led to underestimate the association of area-level deprivation with mortality. In the UK, data on area-level deprivation has been collected routinely since 2000 by using an instrument specifically designed for the purpose, known as the index of multiple deprivation (IMD) [14].

To the best of our knowledge, no previous prospective studies have evaluated the association of IMD with overall and cause-specific mortality in the general population in the UK. The aim of this study is to evaluate the impact of IMD on overall and cause-specific mortality in older British women. In order to present our results in the context of previous research in the area, we conducted a systematic review of prospective studies that examined the association of area-level deprivation and overall and cause-specific mortality.

## Methods

### British Women’s Heart and Health Study

#### Study population

The British Women’s Heart and Health Study (BWHHS) is a prospective cohort study of women aged between 60 and 79 years randomly selected from general practitioner lists from 23 towns across England, Scotland and Wales. Full details of the selection of participants and measurements used in the study have been previously reported [15]. Between April 1999 and March 2001 a total of 4,286 women were interviewed and examined, and completed questionnaires. This study was approved by the London Multi-Centre Research Ethics Committee and Local Research Ethics Committees (Awdurdod Lechyd Bro Taf Health Authority (Wales), Burnley Pendle & Rossendale, County Durham Health Authority, East Cumbria, East Suffolk, Exeter, Fife, Great Yarmouth & Waveney, Harrogate Health Care, Hartlepool Health Care, North Bedfordshire District, North Nottinghamshire Health, North Sefton, North Staffordshire Health, Shropshire, South Humber Health Authority, South west Surrey, Southmead, Wigan & Leigh). All women provided written informed consent.

#### Outcomes

The outcomes of interest were overall and cause-specific mortality from vascular, cancer, respiratory and other causes. Information on cause of death was obtained from the Office for National Statistics and updated until 1st March 2012.

Specific causes of death were coded according to the tenth revision of the International Classification of Diseases (ICDs-10). For analysis, the causes of death were classified as vascular, cancer, respiratory disease and other-causes. Vascular mortality encompasses coronary heart disease, stroke and others. Cancer deaths were classified into two groups: smoking related (deaths from lung, stomach, pancreas, bladder, upper aerodigestive (including oesophagus), kidney, myeloid leukaemia, and liver cancer) and non smoking related (others cancer deaths) [16]. The specific ICD-10 codes used for each cause of mortality are reported in Table **S1**.

#### Area-level deprivation: Index of Multiple Deprivation (IMD)

We used the IMD score released in 2004-2005. Data for the 2004-2005 IMD was gathered from data sources collected in 2001 [17–19], roughly equivalent to the baseline of the BWHHS. The IMD combines weighted scores in seven domains for England and Wales and six domains for Scotland (Table **S2**).

Due to small differences in the composition of the IMD score by country, for the analysis the IMD score from each country (England, Wales and Scotland) was divided into four categories according to differences in the standard deviations (SDs): Category-1 (0 to 1-SD; reference category (least deprived)), Category-2 (1 to 2 SD), Category-3 (2 to 3 SD) and Category-4 (≥3 SD; most deprived). We used the values of SDs based on the overall distribution from each country rather than the one observed in the BWHHS sample. The SD values used were 15.7 for England; 14.3 for Wales; and 16.6 for Scotland. In order to explore the variation over time in the IMD score, we compared the IMD scores of 2004 against 2010 in England, and showed that 85% of the 32,482 Lower Super Output Areas (LSOAs) in England remained in the same IMD category after six years. The areas that showed some changes corresponded to an almost equal number of up or downstream moves (7.3% of the small areas deteriorated and 7.8% of the small areas improved their IMD score) (Figure **S1**).

#### Individual characteristics

The *demographic factors* included age and lifecourse SEP and these were considered as confounders at individual level of the association of IMD with mortality. Other potential confounders at the area-level (e.g. social capital, or built environment features) are not available in the BWHHS. As described previously, lifecourse SEP was measured by combining ten binary items, including six indicators of childhood SEP and four of adulthood SEP [20] In this analysis lifecourse SEP score was calculated for women that responded to six or more items (n=4,083), and was created by combining the binary responses and then dividing the sum by the number of items answered. The average value was then multiplied by ten. A higher score indicates higher level of deprivation across the lifecourse.

The following *health-related behaviours* were considered as *mediators* of effects of area deprivation: physical activity, alcohol consumption, fruit and vegetable intake and smoking. In order to minimize potential residual confounding, we replaced questionnaire-based variable on smoking with the biomarker cotinine [21]. Physical activity was measured by asking women how many hours they spent in the following activities defined as moderate or vigorous physical activity (MVPA) in a typical week during the last year: walking at fairly brisk or fast pace, cycling, heavy gardening and participating in structured exercise [22]. Based on the information, a three-category variable was created: less than 2h per week, 2 to 3h and 3h or more per week of MVPA. Alcohol intake was assessed by the questions: “How many alcoholic drinks do you take during an average week?” and “What type of drink do you usually take?” A British unit of alcohol (approximating 10 g of alcohol) was defined as a half of beer, a single measure of spirits or a glass of wine. We classified women into three groups: non-drinker, moderate drinker (1 to 13 units a week) and heavy drinker (14 or more units a week). Fruit and vegetable intake was obtained through questionnaire, by asking women how often (more than once a day, daily, most days, once or twice a week, less than weekly, or never) they ate fresh fruits and green vegetables. Frequency of consumption was define as: less than twice per day, 2 or 3 times per day and 4 or 5 times per day [23]. Categories of exposure to tobacco using cotinine levels were generated as follows: non-smokers with undetectable second-hand smoke (SHS) exposure (cotinine ≤0.05 ng/ml), non-smokers with detectable SHS exposure (cotinine levels between 0.06 and 15 ng/ml) and current smokers with cotinine levels of 15 ng/ml or more were split into three equal-sized groups [24]. The *biological factors* considered as potential *mediators* of area-level deprivation with mortality were body mass index (BMI), systolic blood pressure (BP), low-density lipoprotein cholesterol (LDL-C) and lung function measured by ratio of forced expiratory volume in 1s to forced vital capacity (FEV1/FVC ratio), all of which are established determinants of major causes of premature mortality and details of measurement of these factors in the BWHHS have been described elsewhere [15]. For analysis systolic BP and LDL-C were treated as continuous variables, BMI was divided into four categories (underweight (<20 kg/m^2^), normal weight (20-24.9 kg/m^2^), overweight (25-29.9 kg/m^2^) and obesity (≥30 kg/m^2^)), and FEV1/FVC ratio was divided in quartiles. We also included self-reported use of statins (British National Formulary (BNF) code 0.12s) and BP lowering medication (BNF codes 02.02.01, 02.02.08, 02.05.01-02.05.06, 02.06.02, 02.04) obtained at baseline as potential mediators.

### Statistical Analysis

To evaluate the association of IMD categories with baseline covariates, we used logistic regression for binary variables, linear regression for continuous variables, and ordered logistic regression for ordinal variables.

Overall and cause-specific mortality rates were calculated by dividing the number of deaths by the person-years of follow-up within of the four IMD categories. Furthermore, we estimated the survival curves of overall and cause-specific mortality according to IMD categories.

We assessed the association between IMD categories and overall and cause-specific mortality using Cox proportional hazards regression models to estimate the hazard ratios (HRs) for each category of IMD, using the least deprived group as the reference group. In addition, we also estimated the HR per one increase in SD of the IMD score. Four cumulative models, using the maximum number of women available for each model, were used to assess the association between IMD and overall and cause-specific mortality: Model-1 was age-adjusted; Model-2 added lifecourse measures of individual deprivation; Models-3 added health-related behaviours (physical activity, alcohol intake, fruit and vegetable intake and cotinine levels); and Model-4 additionally adjusted for biological factors (BMI, systolic BP, LDL-C and FEV _1_/FVC ratio) and self-reported cardiovascular medication. We quantified the percentage of excess risk (in an additive scale) explained by the variables included in models 2 to 4 compared to model-1 using the formula ((HR_Model-1_ - HR_Model-2/3/4_)/(HR_Model-1_ -1.0))x100.

In the analyses of specific cause of death, deaths attributed to other causes of death were treated as censored at the time of death. The proportional hazards assumption was examined by correlating a set of scaled Schoenfeld residuals against a transformation of time; and the influential participants were identified by means of the estimate of DFBETA (estimated change in the coefficient if an individual is removed) [25]. There was no evidence showing that the hazards were not proportional over the follow-up period studied or that there were influential participants.

Although multilevel survival models were not used for the main analysis due to the structure of the data (few women in some LSOAs and low mortality rate), these were conducted and reported for comparison with the standard Cox models. Cox multilevel models were estimated including a LSOA-level random intercept and a Gaussian distribution was postulated for the LSOA-level random effect. Others sensitivity analysis included only women with data on all the covariates (nested models) to explore whether differences in parameter estimates could be attributable to missing values, and the addition of waist-circumference, an independent risk factor of mortality in the elderly [26], to a full adjusted model that also included BMI.

R software version 2.15.0 was used for data analysis in the Cox regression models [27], and for data processing, STATA software version 12 was used.

### Systematic review and meta-analysis of area-level deprivation and cause-specific mortality

Reporting follows the Meta-analysis Of Observational Studies in Epidemiology (MOOSE) Guidelines [28] and the Strengthening the Reporting of Observational Studies in Epidemiology (STROBE) Statement [29].

We identified published studies in general population that evaluated the association of any composite index of area deprivation and overall or cause-specific mortality by searching PubMed until 31st August 2012. Details of the search strategy, selection criteria and data extraction are described in Text **S1**. Additional studies were retrieved from references of identified publications, including meta-analyses and systematic reviews. We used random effect models to pool across studies the relative risks (RRs) for the top-tertile (least deprived; reference group) vs. the bottom-tertile (most deprived; exposure group) of the distribution of area-level deprivation for overall and cause-specific mortality. This was done separately for models with minimal and maximal adjustment. Sub-group analysis (according to type of measure used for area-level deprivation; geographical unit; study design; and study size) was used to explore sources of heterogeneity. Analyses included our findings from the BWHHS and the details of the methodology used for the meta-analysis are described in Text **S1**.

## Results

### British Women’s Heart and Health Study

After excluding one woman with no postcode of residence 4,285 women were included in the present analysis. The proportion of missing data for each variable included in the analysis (median 7.6%; range (7.0% to 9.5%)) is described in Table **S3**. Overall, it was observed that participants in the most deprived areas tended to have a higher proportion of missingness (Table **S3**).

Distribution of potential confounders and mediators across the IMD categories is presented in Tables **1** and **2**. Compared with the women living in the least deprived area, women living in the most deprived area were more deprived through lifecourse, exposed to higher levels of tobacco smoking (second-hand or active smoking), as indicated by the levels of cotinine, and more likely to be physical inactive. In addition, women in the most deprived area were less likely to have a moderate intake of alcohol, and more likely to eat insufficient amount of fruit or vegetable (Table **1**). A positive association of area-level deprivation with BMI and systolic BP was observed, but no major differences were observed for FEV1/FVC ratio, or LDL-C levels (Table **2**).

**Table 1:** Sociodemographic factors and health-related behaviours across categories of the IMD.

		**Categories of IMD**				
**Characteristic**	**Overall**	**C-1 [0 to 1SD**]** (Least Deprived**)	**C-2 [1 to 2SD**]	**C-3 [2 to3SD**]	**C-4[≥3SD**]** (Most Deprived**)	**p-Value^a^**
	**N=4,285**	**n=1,876**	**n=1,458**	**n=604**	**n=347**	
Age (years), mean (SD)	68.9 (5.5)	68.7 (5.5)	68.8 (5.5)	69.0 (5.4)	69.8 (5.6)	≤0.01
Lifecourse SEP score, mean (SD)	4.2 (2.3)	3.4 (2.1)	4.7 (2.2)	5.3 (2.1)	5.5 (2.2)	≤0.001
Physical Activity (MVPA)						
*<2 hr per week*	2,674 (62.4)	1,061 (56.6)	922 (63.2)	430 (71.2)	261 (75.2)	≤0.001
*2-3 hr per week*	435 (10.2)	219 (11.7)	144 (9.9)	51 (8.4)	21 (6.1)	
*≥4 hr per week*	994 (23.2)	535 (28.5)	314 (21.5)	98 (16.2)	47 (13.5)	
Alcohol (units/per-week)						
*0 units/week*	2,015 (47.0)	757 (40.4)	738 (50.6)	335 (55.5)	185 (53.3)	≤0.001
*1-13 units/week*	1,384 (32.3)	706 (37.6)	433 (29.7)	154 (25.5)	91 (26.2)	
*≥14 units/week*	562 (13.1)	307 (16.4)	159 (10.9)	60 (9.9)	36 (10.4)	
Fruit and vegetables intake (times/per-day)						
*<2 times/day*	1,814 (42.3)	718 (38.3)	660 (45.3)	276 (45.7)	160 (46.1)	≤0.001
*2-3 times/day*	1,398 (32.6)	733 (39.1)	414 (28.4)	163 (27.0)	88 (25.4)	
*≥4 times/day*	365 (8,5)	196 (10.4)	109 (7.5)	40 (6.6)	20 (5.8)	
Serum cotinine level (ng/ml)						
*≤0.05 ng/ml*	1,535 (35.8)	897 (47.8)	427 (29.3)	149 (24.7)	62 (17.9)	≤0.001
*0.06-15 ng/ml*	1,822 (42.5)	694 (37.0)	697 (47.8)	272 (45.0)	159 (45.8)	
*15.1-157.0 ng/ml*	157 (3.7)	59 (3.1)	55 (3.8)	25 (4.1)	18 (5.2)	
*157.1-270.6 ng/ml*	157 (3.7)	51 (2.7)	60 (4.1)	26 (4.3)	20 (5.8)	
*≥270.7 ng/ml*	157 (3.7)	48 (2.6)	53 (3.6)	28 (4.6)	28 (8.1)	

All values are numbers (and percent), except where noted.

Note: IMD categories were based on the SD from the overall score from each country (SD by country: England= 15.7, Wales= 14.3 and Scotland= 16.6).

^a^ Logistic regression for binary variables, linear regression for continuous variables, and ordered logistic regression for ordinal variables.

IMD, index of multiple deprivation; SD, standard deviation; SEP, socioeconomic position; MVPA, moderate or vigorous physical activity

**Table 2:** Biological factors and self-reported use of cardiovascular medication across categories of the IMD.

		**Categories of IMD**				
**Characteristic**	**Overall**	**C-1 [0 to 1SD**]** (Least Deprived**)	**C-2 [1 to 2SD**]	**C-3 [2 to3SD**]	**C-4[≥3SD**]** (Most Deprived**)	**p-Value^a^**
	**N=4,285**	**n=1,876**	**n=1,458**	**n=604**	**n=347**	
BMI (Kg/m^2^)						
*<20 kg/m* ^*2*^	112 (2.6)	46 (2.5)	42 (2.9)	13 (2.2)	11 (3.2)	≤0.001
*20-24.9 kg/m* ^*2*^	1,151 (26.9)	600 (32.0)	347 (23.8)	128 (21.2)	76 (21.9)	
*25-29.9 kg/m* ^*2*^	1,645 (38.4)	772 (41.1)	549 (37.6)	211 (34.9)	113 (32.6)	
*≥30 kg/m* ^*2*^	1,049 (24.5)	383 (20.4)	398 (27.3)	169 (28.0)	99 (28.5)	
Systolic BP (mmHg), mean (SD)	147.1 (25.2)	146.0 (24.5)	147.8 (25.9)	147.9 (24.8)	149.1 (26.0)	0.073
LDL-c (mmol/l), mean (SD)	4.1 (1.1)	4.1 (1.1)	4.2 (1.1)	4.2 (1.1)	4.1 (1.1)	0.620
FEV_1_/FVC ratio						
*<0.66*	983 (22.9)	421 (22.4)	341 (23.4)	137 (22.7)	84 (24.2)	0.694
*0.66-0.71*	983 (22.9)	471 (25.1)	325 (22.3)	120 (19.9)	67 (19.3)	
*0.72-0.76*	984 (23.0)	449 (23.9)	340 (23.3)	121 (20.0)	74 (21.3)	
*≥0.77*	982 (22.9)	443 (23.6)	319 (21.9)	147 (24.3)	73 (21.0)	
BP lowering medication use						
*No*	2,554 (59.6)	1,189 (63.4)	856 (58.7)	324 (53.6)	185 (53.3)	0.208
*Yes*	1,432 (33.4)	623 (33.2)	489 (33.5)	204 (33.8)	116 (33.4)	
Statins medication use						
*No*	3,691 (86.1)	1,706 (90.9)	1,221 (83.7)	487 (80.6)	277 (79.8)	≤0.01
*Yes*	295 (6.9)	106 (5.7)	124 (8.5)	41 (6.8)	24 (6.9)	

All values given as n (percent), except where noted.

Note: IMD categories were based on the SD from the overall score from each country (SD by country: England= 15.7, Wales= 14.3 and Scotland= 16.6).

^a^ Logistic regression for binary variables, linear regression for continuous variables, and ordered logistic regression for ordinal variables.

IMD, index of multiple deprivation; SD, standard deviation; BMI, body mass index; BP, blood pressure; LDL-c, low-density lipoprotein cholesterol; FEV _1_/FVC, forced expiratory volume in one s/ forced vital capacity

**Figure 1:**
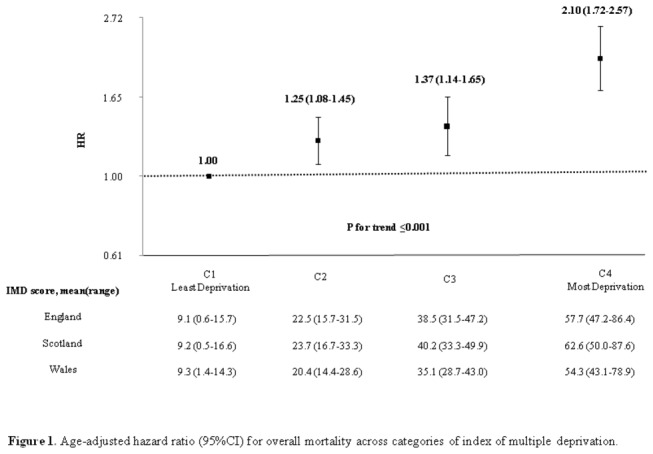
Age-adjusted hazard ratio (95%CI) for overall mortality across categories of index of multiple deprivation. The vertical axis is plotted in the log-scale. The distances between categories in the horizontal axis correspond to the mean values for index of multiple deprivation (IMD) in England. IMD categories were based on the standard deviation (SD) from the overall score from each country (SD by country: England= 15.7, Wales= 14.3 and Scotland= 16.6). Numbers above each box indicate the corresponding hazard ratios and 95% confidence intervals. The mean and the range were calculated from the overall score from each country by IMD categories.

After a median follow up of 11.6 years (range 11.0-12.1) a total of 1,009 (23.5%) women died. Of these deaths, 343 (34.0%) were attributable to vascular disease, 327 (32.4%) to cancer, 127 (12.6%) to respiratory disease and 212 (21%) to other-causes. Table **3** shows that as level of area-level deprivation increased, the rates of overall mortality rose gradually, with women in the highest IMD category having a doubled mortality rate compared with women in the bottom IMD category (39 vs. 17.9 per 1000 person-year, respectively). After adjustment for age, the positive dose-response association between IMD categories and overall mortality remained robust (P-value for linear trend <0.001; Figure **1**). Similar dose-response associations were shown for the cause-specific mortality rates (Table **3**). However, it is important to note that the magnitude of association differed according to the cause of death. Respiratory deaths showed the greatest increase (HR=1.54; 95% CI: 1.31-1.80) per 1-SD increase in IMD score, follow by vascular deaths and other-causes (HR=1.29; 95% CI: 1.16-1.43 and HR=1.23; 95% CI: 1.08-1.41, respectively). The smallest increase was found for cancer deaths (HR=1.13; 95% CI: 1.01-1.23) (Table **3**).

**Table 3:** Number of deaths (N) and rate per 1,000 person-year (95%CI) of overall and cause-specific mortality across categories of the IMD.

	**C-1 [0 to 1SD**]** (Least Deprived**)		**C-2 [1 to 2SD**]		**C-3 [2 to3SD**]		**C-4[≥3SD**]** (Most Deprived**)		**Hazard ratio (95%CI**)** per 1-SD increase**
**Cause of death**	**n=1,876**		**n=1,458**		**n=604**		**n=347**		
	**N**	**Rate (95%CI**)	**N**	**Rate (95%CI**)	**N**	**Rate (95%CI**)	**N**	**Rate (95%CI**)	**Age-Adjusted**
**Vascular disease:**									
Coronary heart disease	59	2.9 (2.2-3.8)	47	3.0 (2.2-4.0)	25	3.9 (2.5-5.7)	24	7.1 (4.5-10.6)	1.34 (1.15-1.55)
Stroke	37	1.8 (1.3-2.5)	41	2.6 (1.9-3.5)	14	2.2 (1.2-3.6)	9	2.7 (1.2-5.1)	1.10 (0.90-1.34)
Other vascular	20	1.0 (0.6-1.5)	40	2.5 (1.8-3.5)	15	2.3 (1.3-3.8)	12	3.5 (1.8-6.2)	1.43 (1.18-1.74)
All vascular	116	5.7 (4.7-6.9)	128	8.1 (6.8-9.7)	54	8.4 (6.3-10.9)	45	13.3 (9.7-17.8)	1.29 (1.16-1.43)
**Cancers:**									
Cancers related to smoking	58	2.9 (2.2-3.7)	44	2.8 (2.0-3.8)	24	3.7 (2.4-5.5)	17	5.0 (2.9-8.0)	1.17 (0.99-1.38)
Cancers not related to smoking	78	3.9 (3.0-4.8)	65	4.1 (3.2-5.3)	19	2.9 (1.8-4.6)	22	6.5 (4.1-9.8)	1.10 (0.94-1.27)
All cancer	136	6.7 (5.67.9)	109	6.9 (5.7-8.4)	43	6.7 (4.8-9.0)	39	11.5 (8.2-15.8)	1.13 (1.01-1.23)
**Respiratory disease:**	31	1.5 (1.0-2.2)	46	2.9 (2.1-3.9)	30	4.7 (3.1-6.6)	20	5.9 (3.6-9.1)	1.54 (1.31-1.80)
**Other-causes**	79	3.9 (3.1-4.9)	71	4.5 (3.5-5.7)	34	5.3 (3.7-7.4)	28	8.3 (5.5-12.0)	1.23 (1.08-1.41)
**All-causes**	362	17.9 (16.1-19.8)	354	22.5 (20.2-25.0)	161	25.0 (21.3-29.1)	132	39.0 (32.6-46.3)	1.25 (1.18-1.33)

Note: IMD categories were based on the SD from the overall score from each country (SD by country: England= 15.7, Wales= 14.3 and Scotland= 16.6). IMD, index of multiple deprivation; SD, standard deviation.

IMD, index of multiple deprivation; SD, standard deviation, CI: confidence interval

After we added lifecourse measures of individual deprivation to an age-adjusted model, the point estimate of the HR per 1-SD increase in the IMD score for all the cause-specific mortality decreased by 20% to 24%, with exception of other-causes that remained largely unchanged (**Model 2;** Table **4**). Compared with an age-adjusted model, the association of IMD on all-cancers thought the smallest in magnitude, disappeared after inclusion of lifecourse SEP score in the model. Adjustment for health behaviours (considered as mediators) resulted in a 44% and 48% reduction in the point estimate of the HR for respiratory and other-causes, with no changes for vascular deaths. Further inclusion of potential mediators such as biological factors and use of cardiovascular medications in the previous model produced an almost negligible reduction in the HR for all the outcomes evaluated (**Model 4;** Table **4**).

**Table 4:** Hazard Ratio (95%CI) of cause-specific death per 1-SD increase in the IMD score.

	**Model 1 (n=4,285**)		**Model 2 (n=4,083**)		**Model 3 (n=3,071**)		**Model 4 (n=2,912**)	
**Cause of death**	No. of deaths	Adjusted for age	No. of deaths	Adjusted for Model 1 variables plus Lifecourse SEP score	No. of deaths	Adjusted for Model 2 variables plus health behaviours^a^	No. of deaths	Adjusted for Model 3 variables plus biological factors^b^ and CVD medication^c^
**Vascular disease**	343	1.29 (1.16-1.43)	319	1.22 (1.09-1.37)	202	1.22 (1.05-1.42)	187	1.22 (1.03-1.44)
**Cancers**	327	1.13 (1.01-1.23)	306	1.10 (0.97-1.24)	219	1.04 (0.89-1.22)	207	1.08 (0.92-1.27)
**Respiratory disease**	127	1.54 (1.31-1.80)	120	1.43 (1.20-1.71)	71	1.30 (1.02-1.66)	66	1.27 (0.97-1.67)
**Other-causes**	212	1.23 (1.08-1.41)	198	1.24 (1.07-1.44)	129	1.12 (0.92-1.36)	120	1.09 (0.88-1.35)
**All-causes**	1,009	1.25 (1.18-1.33)	943	1.21 (1.13-1.30)	621	1.15 (1.05-1.25)	580	1.15 (1.04-1.26)

Note: IMD categories were based on the SD from the overall score from each country (SD by country: England= 15.7, Wales= 14.3 and Scotland= 16.6).

^a^ Health-related behaviours included physical activity, alcohol intake, fruit and vegetables intake and concentrations of cotinine.

^b^ Biological factors included BMI, systolic BP, LDL-c and FEV _1_/FVC ratio.

^c^ CVD medication include self-reported statins and BP lowering medication.

IMD, index of multiple deprivation; SD, standard deviation; CI, confidence interval; SEP, socioeconomic position; BMI, body mass index; BP, blood pressure; LDL-c, low-density lipoprotein cholesterol; FEV _1_/FVC, forced expiratory volume in one s/ forced vital capacity ratio; CVD, cardiovascular disease.

**Figure 2:**
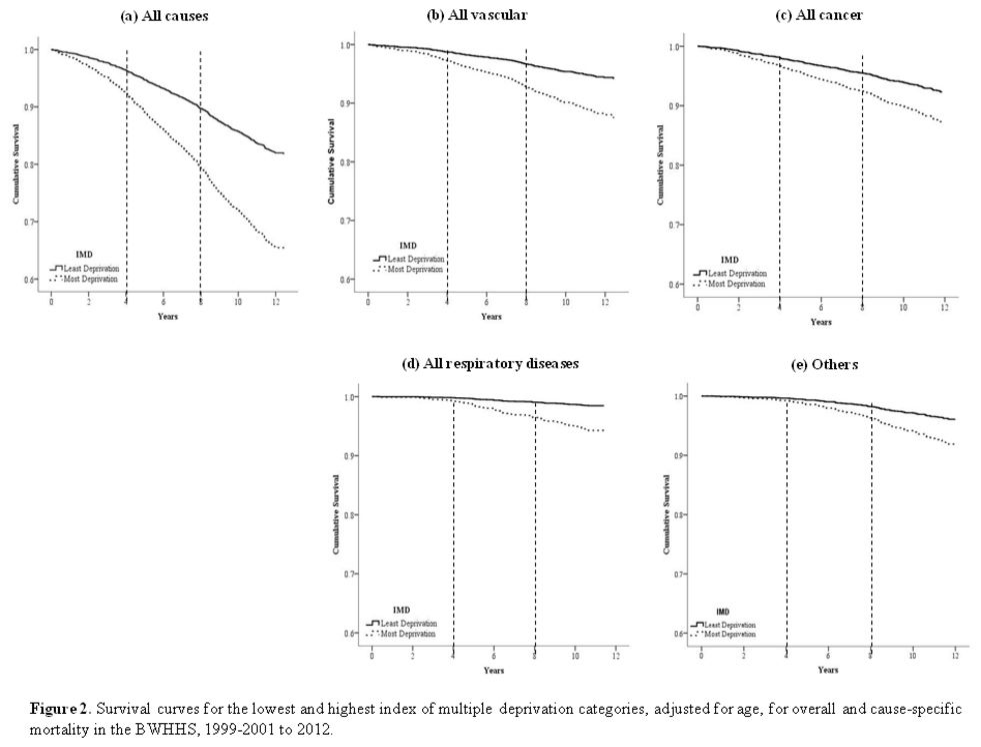
Survival curves for the lowest and highest index of multiple deprivation categories, adjusted for age, for overall and cause-specific mortality in the BWHHS, 1999-2001 to 2012. (a) All-cause, (b) all vascular, (c) all cancer, (d) all respiratory disease and (e) others. The Y-axes were truncated to 0.6 for a better visualization. The dashed lines divide the graphs into periods of four years.

Figure **2** shows the age-adjusted cumulative survival between the top and bottom IMD categories and indicates a clear separation of the survival curves for overall mortality within the first few years of follow-up, which then increase in a proportional way over time. Survival curves for cause-specific mortality showed that vascular disease and cancers were responsible for the early separation of the curves, while the respiratory disease and other-causes of death started to contribute predominantly after four years of follow-up.

A series of sensitivity analyses showed the robustness of the previous results. First, results from multilevel Cox regression models were virtually identical to those from standard Cox regression models (Table **S4**). Second, multivariate models with complete data on all variables provided results that did not differ substantially from those obtained in the main analyses (Table **S5**). Third, addition to model-4 of waist circumference, a strong predictor of death in elderly, did not alter the effects of area-level deprivation on mortality (Table **S6**).

### Systematic Review

**Figure 3:**
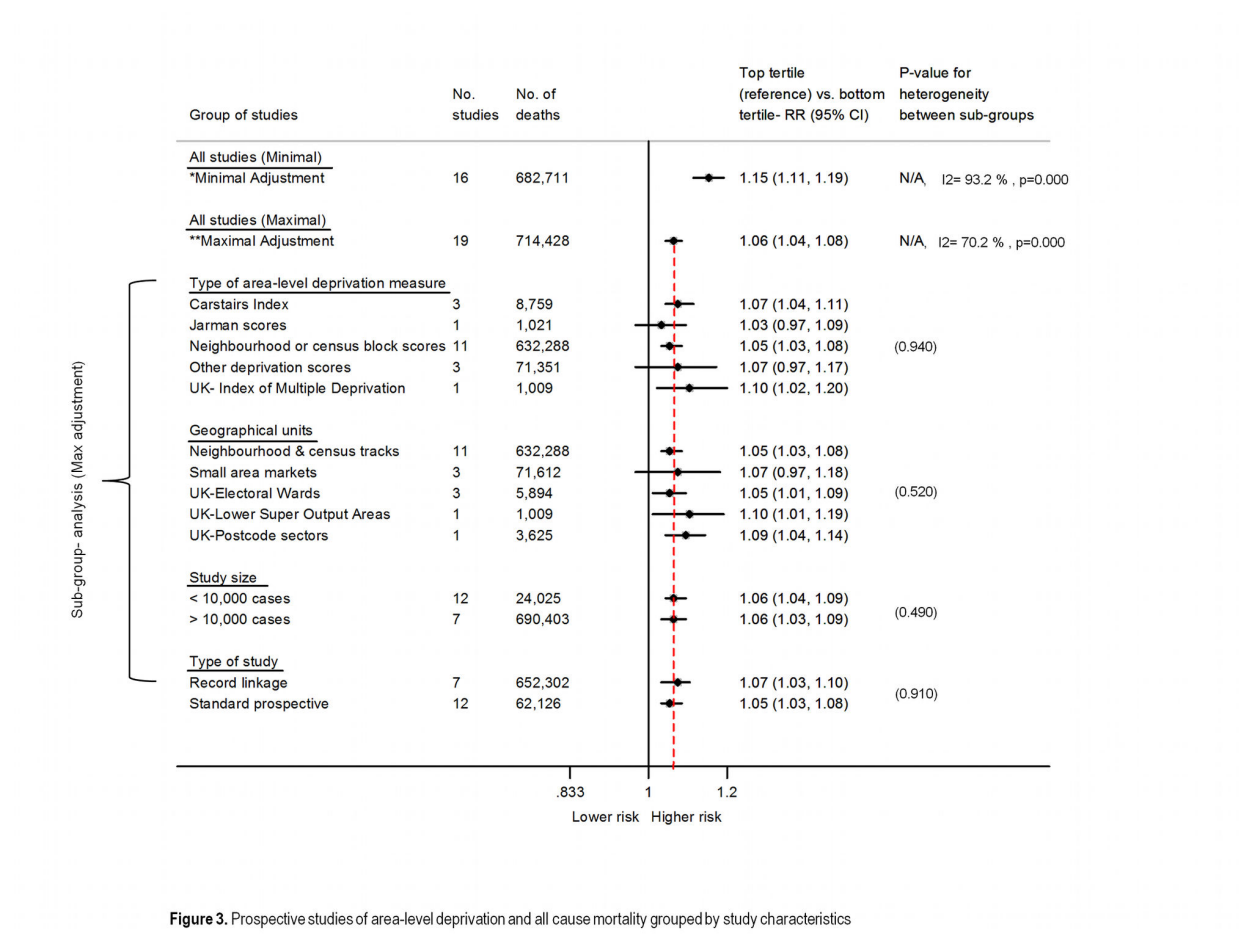
Prospective studies of area-level deprivation and all-cause mortality grouped by study characteristics. For degree of adjustment from individual studies please see Table **S7, S8** and Figures **S3** and **S4**.

In addition to the BWHHS, a total of 20 prospective studies were included, of these eleven were standard prospective studies and nine were prospective record-linkage studies (Figure **S2**). Characteristics of studies included in the systematic review are reported in Tables **S7** and **S8**. The outcome most commonly evaluated in published studies was all-cause mortality (n=18), followed by vascular (n=7), cancer (n=4) and respiratory mortality (n=1). None of the published studies included all major causes of death as we did (Table **S9**). Measures of individual deprivation were limited to variables that capture deprivation in middle-age/elderly, and only four out of 20 studies included all three most common measures (education, income and occupation) of individual deprivation (Table **S10**). Degree of adjustment for the most complete model was highly variable by study, with only five studies including area-level confounders and only two studies adjusting for all four health-related behaviours as we did, additional studies provided incomplete adjustment for health-related behaviours (Table S7, S8 and S10). The summary RR of area-level deprivation with total mortality changed substantially from a minimal to a maximal adjusted model 1.15 (95% CI: 1.11, 1.19) to 1.06 (95% CI: 1.04-1.08), respectively (Figures **S3** and **S4**). The summary RR from maximal adjustment models did not differ considerably according to the measure of area-level deprivation used, geographical unit, study design or study size (Figure **3**). The summary RR from the maximal adjustment model, including the results from our study (N=21 studies), for cause-specific mortality was 1.09 (95% CI: 1.04-1.14) for vascular disease, 1.05 (95% CI: 1.00-1.11) for cancers and 1.09 (95% CI: 0.93-1.27) for respiratory disease. For details of meta-analysis results according to level of adjustment and type of prospective study (standard vs. record-linkage) see Table **S11**.

## Discussion

The present study showed that increasing levels of deprivation at area-level were associated with an increase in risk of overall mortality. The association appears to be independent of individual deprivation through lifecourse and largely, but not entirely, mediated by health-related behaviours. Results from the systematic review provided further support to these statements and showed that the risk of overall mortality for those in the top-third of the distribution of the area-level deprivation reduced from 15% to a 6% after accounting for health-related behaviours and biological factors.

Interestingly, analysis in the BWHHS on cause-specific mortality showed that the association of area-level deprivation (measured by IMD) rather than cause-specific was observed with all major groups of mortality. However we did observe a variation in the magnitude of the association, with respiratory disease having the highest hazard, followed by vascular disease and other-causes, while cancer deaths had the smallest one. Though the evidence from published studies on cause-specific mortality was limited, they supported the existence of a greater association with respiratory disease, follow by vascular disease.

The statistical adjustment at BWHHS indicated that measures of health-related behaviours available to us *mediate* the association of area-level deprivation with cancer mortality, but these measures were insufficient to explain the association with respiratory and vascular deaths. This residual effect could be due to imprecise measures of health-related behaviours, particularly the measures of physical activity and fruit and vegetable consumption used in this study that relied on self-reports [30]. Another possibility is that the residual effect could be explained by unmeasured mediators, such as environmental factors (e.g. air quality), which may be more relevant to respiratory deaths. Interestingly, adjustment for lung function, a known risk factor of respiratory deaths, had a negligible effect on the association of area-level deprivation with respiratory deaths when health-related behaviours were controlled for [31].

Evidence from the systematic review was concordant with our results, and showed that the association of area-level deprivation with overall and cause-specific mortality was largely accounted for by health-related behaviours and biological factors as indicated by the marginal RRs from the most adjusted models (all-causes mortality 1.06 (95% CI: 1.04-1.08); vascular 1.09 (95% CI: 1.04-1.14); cancer 1.05 (95% CI: 1.00-1.11); and respiratory 1.09 (95%CI: 0.93-1.27)) (Table **S11**). Nonetheless, since adjustment for health-related behaviours in the published studies was largely incomplete and based on self-report measures, it is likely that the small residual risk could be entirely mediated by these factors.

The present study has some strengths and limitations. Although, we limited our systematic review to studies that used composite measures of area deprivation that intended to capture the multidimensional nature of this exposure, such instruments may be insufficient to fully characterise the area deprivation, and result in attenuation of the observed associations with mortality. This is of relevance, if we considered that only the BWHHS used an instrument (IMD) designed with the specific purpose of measuring deprivation at area-level. Interestingly, results from our meta-analysis did not show a clear difference in the association of area-level deprivation with mortality according to the measure of area-level deprivation used. On the other hand, measurement error in characterising lifecourse *individual deprivation*, primarily from published studies that only included some aspects of adulthood deprivation could lead to residual confounding by individual deprivation [32]. Furthermore, area-level confounders (e.g. built environment or social capital) that tend to be associated with area deprivation and with mortality in some studies [33,34], were rarely included in published studies. Though, this could be due to lack of access of such measures as in the BWHHS.

Another weakness of the BWHHS analysis (as well from published studies) is the missingness in the covariates, given that the level of missingness tended to be differential according to the level of area deprivation. However, a sensitivity analysis in which only the BWHHS participants with complete data were included provided almost identical results, suggesting that missing data are unlikely to invalidate our findings. It is important to note that available evidence to date on the association of area-level deprivation with mortality is derived from high-income countries, thus the findings from our systematic review may not be applicable to low and middle-income countries.

## Conclusions

If the association of area-level deprivation with mortality is causal, it suggests that deprivation in an area could lead to avoidable mortality of residents regardless of their individual level of deprivation. However, the magnitude of the percentage of excess risk, derived from the age-sex adjusted model is small-to-moderate (15%), but may be an under-estimate. This indicates that, in theory, further gains in health are possible through interventions aiming to diminish the levels of deprivation in the community or neighbourhood. Identification and evaluation of such interventions, however, is challenging [35]. Although, the association between area deprivation and mortality is mainly mediated by health-related behaviours, policies aiming to modify behavioural factors (e.g. smoking ban and taxation of cigarettes) that reduce national levels of consumption [36] may be less successful in deprived areas [37]. Increasing access to cardiovascular medications known to reduce premature mortality [38] would be another strategy but, if those are delivered using standard health-care routes, is also prone to differential impact in deprived areas [39].

## Supporting Information

Checklist S1 **PRISMA checklist.**
(DOC)

Figure S1 **Scatter plot of the mean scores of IMD in 2004 against IMD in 2010 for 32,482 LSOAs from England.**
(DOC)

Figure S2 **Study selection process for systematic review.**
(DOC)

Figure S3 **Meta-analysis of prospective studies (minimal adjustments) evaluating the association of area-level deprivation with all-cause mortality.**
(DOC)

Figure S4 **Meta analysis of prospective studies (maximal adjustment) of area-level deprivation and all-cause mortality.**
(DOC)

Table S1 **ICD-10 codes used to identify cause of death.**
(DOC)

Table S2 **Domains of the IMD available at LSOA level by country.**
(DOC)

Table S3 **Number (percent) of women with missing data across categories of the IMD.**
(DOC)

Table S4 **Hazard Ratio and 95% confidence interval per 1-SD increase in the IMD score, multilevel Cox regression versus standard Cox regression.**
(DOC)

Table S5 **Hazard Ratio (95% CI) of cause-specific death per 1-SD increase in the IMD score among participants with complete information in all co-variables.**
(DOC)

Table S6 **Change in the hazard ratio (95% CI) of cause-specific death per 1-SD increase of IMD score after adding waist circumference to the model-4.**
(DOC)

Table S7 **Summary details of published standard prospective studies investigating the association of area-level deprivation and overall or cause-specific mortality.**
(DOC)

Table S8 **Summary details of published record linkage studies investigating the association of area-level deprivation and overall or cause-specific mortality.**
(DOC)

Table S9 **Reporting specific-causes of mortality from studies included in the systematic review.**
(DOC)

Table S10 **Reporting individual level socioeconomic position and area-level confounder from studies included in the systematic review.**
(DOC)

Table S11 **Summary relative risks (95% CI) for area-level deprivation and specific health outcomes derived from standard prospective and record-linkage studies.**
(DOC)

Text S1 **Supporting Methods; Supporting Results; Supporting References.**
(DOC)
