# Supplementary material for: Area-Level Deprivation and Overall and Cause-Specific Mortality: 12 Years’ Observation on British Women and Systematic Review of Prospective Studies
Source: PLoS One. 2013 Sep 24;8(9):e72656. doi: 10.1371/journal.pone.0072656 (PMC3782490; doi:10.1371/journal.pone.0072656)
Supplement: Figure S1 — Scatter plot of the mean scores of IMD in 2004 against IMD in 2010 for 32,482 LSOAs from England. (DOC) [file pone.0072656.s002.doc]

**Figure S1.** Scatter plot of the mean scores of index of multiple deprivation (IMD) in 2004 against IMD in 2010 for 32,482 lower super output areas (LSOAs) from England. Eighty-five percent of the 32,482 LSOAs remained in the same IMD category used in the analysis for the BWHHS study after 6 years. The areas that showed some changes corresponded to an almost equal number of up or downstream moves (7.3% of the small areas deteriorated and 7.8% of the small areas improved their IMD score)
