# Supplementary figures and images for: Area-Level Deprivation and Overall and Cause-Specific Mortality: 12 Years’ Observation on British Women and Systematic Review of Prospective Studies
Source: PLoS One. 2013 Sep 24;8(9):e72656. doi: 10.1371/journal.pone.0072656 (PMC3782490; doi:10.1371/journal.pone.0072656)

**Figure S2.** Study selection process for systematic review


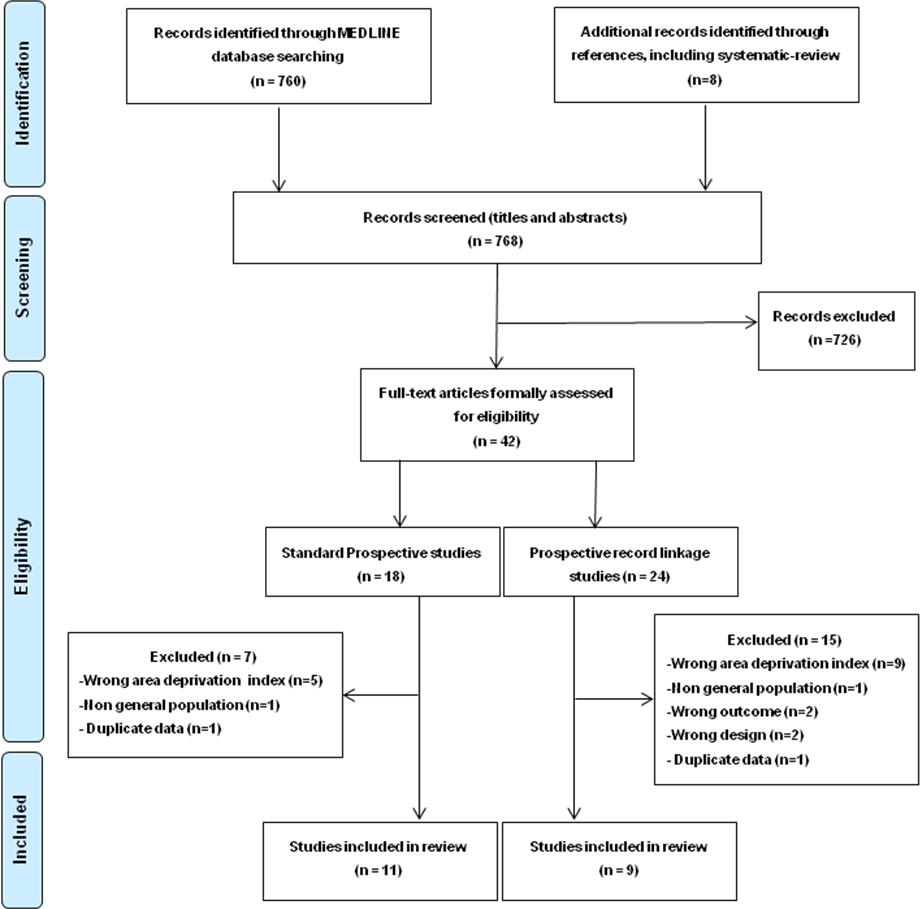

Supplement: Figure S2 — Study selection process for systematic review. (DOC) [file pone.0072656.s003.doc]
