# Supplementary material for: Area-Level Deprivation and Overall and Cause-Specific Mortality: 12 Years’ Observation on British Women and Systematic Review of Prospective Studies
Source: PLoS One. 2013 Sep 24;8(9):e72656. doi: 10.1371/journal.pone.0072656 (PMC3782490; doi:10.1371/journal.pone.0072656)
Supplement: Figure S3 — Meta-analysis of prospective studies (minimal adjustments) evaluating the association of area-level deprivation with all-cause mortality. (DOC) [file pone.0072656.s004.doc]

**Figure S3**. Meta-analysis of prospective studies (minimal adjustments) evaluating the association of area-level deprivation with all-cause mortality. The relative risks correspond to the least adjusted model published by individual studies. Degree of adjustment: + adjustment for age and sex, ++ adjustment for age, sex and/or race and other minimal adjustments.

Studies classified as standard prospective are in webreferences 3, 5, 9, 19, 11, 18, 2, 7, 20 and 17 and those as record linkage in webreferences 6, 12, 15, 1 and 10 in **Text S1**.
