# Supplementary material for: Area-Level Deprivation and Overall and Cause-Specific Mortality: 12 Years’ Observation on British Women and Systematic Review of Prospective Studies
Source: PLoS One. 2013 Sep 24;8(9):e72656. doi: 10.1371/journal.pone.0072656 (PMC3782490; doi:10.1371/journal.pone.0072656)
Supplement: Figure S4 — Meta analysis of prospective studies (maximal adjustment) of area-level deprivation and all-cause mortality. (DOC) [file pone.0072656.s005.doc]

**Figure S4.** Meta-analysis of prospective studies (maximal adjustment) of area-level deprivation and all-cause mortality. Degree of adjustment: + adjustment for sex, age and/or race and ethnicity; ++ for these plus SEP (income, education, housing tenure, state poverty level, financial pressure); +++ for these plus health related behaviours (smoking, alcohol use, physical activity or diet); ++++ for these plus biological factors (Chronic disease, cholesterol, blood pressure, angina, ECG ischemia)

Studies classified as standard prospective are in webreferences 5, 7, 9, 19, 20, 2, 4, 17, 3, 11 and 18 and those as record linkage in webreferences 1, 6, 8, 10, 15, 12 and 13 in **Text S1**.
