# Supplementary material for: Area-Level Deprivation and Overall and Cause-Specific Mortality: 12 Years’ Observation on British Women and Systematic Review of Prospective Studies
Source: PLoS One. 2013 Sep 24;8(9):e72656. doi: 10.1371/journal.pone.0072656 (PMC3782490; doi:10.1371/journal.pone.0072656)
Supplement: Table S1 — ICD-10 codes used to identify cause of death. (DOC) [file pone.0072656.s006.doc]

**Table S1. ICD-10 codes used to indentify cause of death**

| **Cause of death** | **ICD-10 code** |
| --- | --- |
| ***Vascular disease:*** | I00-I99 |
| Coronary heart disease | I20-I25 |
| Stroke | I60-I69 |
| Other vascular | I00-I19, I26-I59, I70-I99 |
| ***Cancers:*** | C00-C97 |
| *Cancers related to smoking:* |  |
| Lung | C33-C34 |
| Stomach | C16 |
| Pancreas | C25 |
| Bladder | C67 |
| Upper aerodigestive | C00-C15 |
| Kidney | C64-C66, C68 |
| Myeloid leukaemia | C92 |
| Liver | C22-C24 |
| ***Respiratory disease*** | J00-J99 |

Note: Other causes of death were remaining ICD-10 codes

ICD, International Classification of Diseases
