# Supplementary material for: Area-Level Deprivation and Overall and Cause-Specific Mortality: 12 Years’ Observation on British Women and Systematic Review of Prospective Studies
Source: PLoS One. 2013 Sep 24;8(9):e72656. doi: 10.1371/journal.pone.0072656 (PMC3782490; doi:10.1371/journal.pone.0072656)
Supplement: Table S2 — Domains of the IMD available at LSOA level by country. (DOC) [file pone.0072656.s007.doc]

**Table S2. Domains of the IMD available at LSOA level by country**

| **Domains** | **Country and year of publication of IMD score (number of women in the study)** | | |
| --- | --- | --- | --- |
|  | **England 2004 (n=3,568)** | **Scotland 2004 (n=546)** | **Wales 2005 (n=171)** |
| Income | √ | √ | √ |
| Employment | √ | √ | √ |
| Health (and disability for England) | √ | √ | √ |
| Education, skills, training | √ | √ | √ |
| Housing | √^a^ | √ | √ |
| Geographical access to services |  | - | √ |
| Geographical access and telecommunications | - | √ | - |
| Living environment | √ | - | - |
| Physical environment | - | - | √ |
| Crime | √ | - | - |

^a^Housing and geographical access to services are combined in England

IMD, index of multiple deprivation; LSOA, lower layer super output areas
