# Supplementary material for: Area-Level Deprivation and Overall and Cause-Specific Mortality: 12 Years’ Observation on British Women and Systematic Review of Prospective Studies
Source: PLoS One. 2013 Sep 24;8(9):e72656. doi: 10.1371/journal.pone.0072656 (PMC3782490; doi:10.1371/journal.pone.0072656)
Supplement: Table S3 — Number (percent) of women with missing data across categories of the IMD. (DOC) [file pone.0072656.s008.doc]

**Table S3**. Number (percent) of women with missing data across categories of the IMD. All values are numbers (and percent).

|  | **Overall**  **(N=4,285)** | **Categories of IMD score** | | | |
| --- | --- | --- | --- | --- | --- |
|  |  | **Category-1**  **[0 to 1SD]**  **(Least Deprived)**  **n=1,876** | **Category-2**  **[1 to 2SD]**  **n=1,458** | **Category-3**  **[2 to3SD]**  **n=604** | **Category-4**  **[≥3SD]**  **(Most Deprived)**  **n=347** |
| **Characteristic** |  |  |  |  |  |
| Age (years) | - | - | - | - | - |
| Lifecourse SEP score<6^a^ | 202 (4.7) | 69 (3.7) | 81 (5.6) | 29 (4.8) | 23 (6.6) |
| Physical Activity (MVPA) | 182 (4.2) | 61 (3.2) | 78 (5.3) | 25 (4.1) | 18 (5.2) |
| Alcohol intake (units/per-week) | 324 (7.6) | 106 (5.6) | 128 (8.8) | 55 (9.1) | 35 (10.1) |
| Fruit and vegetables intake (times/per-day) | 708 (16.5) | 229 (12.2) | 275 (18.9) | 125 (20.7) | 79 (22.8) |
| Serum cotinine (ng/ml) | 457 (10.7) | 127 (6.8) | 166 (11.4) | 104 (17.2) | 60 (17.3) |
| BMI (kg/m2) | 328 (7.6) | 75 (4.0) | 122 (8.4) | 83 (13.7) | 48 (13.8) |
| Systolic BP (mmHg) | 321 (7.5) | 67 (3.6) | 121 (8.3) | 86 (14.2) | 47 (13.5) |
| LDL-C (mmol/l) | 566 (13.2) | 170 (9.1) | 204 (14.0) | 126 (20.9) | 66 (19.0) |
| FEV_1_/FVC ratio | 353 (8.2) | 92 (4.9) | 133 (9.1) | 79 (13.1) | 49 (14.1) |
| BP medication use | 299 (7.0) | 64 (3.4) | 113 (7.7) | 76 (12.6) | 46 (13.3) |
| Statins medication use | 299 (7.0) | 64 (3.4) | 113 (7.7) | 76 (12.6) | 46 (13.3) |

Note: IMD categories were based on the SD from the overall score by country (SD by country were England: 15.7, Wales: 14.3 and Scotland: 16.6)

IMD, index of multiple deprivation; SD, standard deviation; SEP, socioeconomic position; MVPA, moderate or vigorous physical activity; BMI, body mass index; BP, blood pressure; LDL-c, low-density lipoprotein cholesterol; FEV_1_/FVC, forced expiratory volume in 1 s/ forced vital capacity ratio.

^a^Women that had answered to less than 6 socioeconomic indicators
