# Supplementary material for: Area-Level Deprivation and Overall and Cause-Specific Mortality: 12 Years’ Observation on British Women and Systematic Review of Prospective Studies
Source: PLoS One. 2013 Sep 24;8(9):e72656. doi: 10.1371/journal.pone.0072656 (PMC3782490; doi:10.1371/journal.pone.0072656)
Supplement: Table S4 — Hazard Ratio and 95% confidence interval per 1-SD increase in the IMD score, multilevel Cox regression versus standard Cox regression. (DOC) [file pone.0072656.s009.doc]

**Table S4.** Hazard Ratio and 95% Confident Intervals per 1-SD increase in the IMD score: multilevel Cox regression^a^ versus standard Cox regression.

|  | **Model 1**  **(n=4,285)** | | **Model 2**  **(n=4,083)** | | **Model 3**  **(n=3,071)** | | **Model 4**  **(n=2,912)** | |
| --- | --- | --- | --- | --- | --- | --- | --- | --- |
| **Cause of death** | Standard Cox Regression | Multilevel Cox Regression | Standard Cox  Regression | Multilevel Cox Regression | Standard Cox  Regression | Multilevel Cox Regression | Standard Cox  Regression | Multilevel Cox Regression |
| **Vascular** | 1.29  (1.16-1.43) | 1.30  (1.17-1.44) | 1.22  (1.09-1.37) | 1.23  (1.09-1.38) | 1.22  (1.05-1.42) | 1.22  (1.04-1.43) | 1.22  (1.03-1.44) | 1.22  (1.02-1.45) |
| **Cancers** | 1.13  (1.01-1.23) | 1.13  (1.01-1.26) | 1.10  (0.97-1.24) | 1.10  (0.97-1.24) | 1.04  (0.89-1.22) | 1.04  (0.89-1.22) | 1.08  (0.92-1.27) | 1.08  (0.92-1.27) |
| **Respiratory** | 1.54  (1.31-1.80) | 1.54  (1.31-1.80) | 1.43  (1.20-1.71) | 1.43  (1.19-1.71) | 1.30  (1.02-1.66) | 1.28  (0.98-1.66) | 1.27  (0.97-1.67) | 1.26  (0.94-1.67) |
| **Other causes** | 1.23  (1.08-1.41) | 1.23  (1.08-1.41) | 1.24  (1.07-1.44) | 1.24  (1.08-1.44) | 1.12  (0.92-1.36) | 1.12  (0.92-1.36) | 1.09  (0.88-1.35) | 1.09  (0.88-1.35) |
| **All causes** | 1.25  (1.18-1.33) | 1.26  (1.18-1.34) | 1.21  (1.13-1.30) | 1.21  (1.13-1.30) | 1.15  (1.05-1.25) | 1.15  (1.05-1.25) | 1.15  (1.04-1.26) | 1.15  (1.04-1.26) |

Note: IMD categories were based on the SD from the overall score by country (SD by country were England: 15.7, Wales: 14.3 and Scotland: 16.6)

^a^LSOAs were used as random effects

IMD, index of multiple deprivation; SD, standard deviation; SEP, socioeconomic position; CVD, cardiovascular disease; BMI, body mass index; FEV1/FVC, forced expiratory volume in 1 s/ forced vital capacity ratio; LSOAs, lower layer super output areas

Model 1: minimal adjusted (age)

Model 2: adjusted for Model 1 variables + lifecourse SEP score

Model 3: adjusted for Model 2 variables + health behaviours (physical activity, alcohol intake, fruit and vegetable intake and concentrations of cotinine)

Model 4: adjusted for Model 3 variables + CVD biomarkers (BMI, SBP, LDL-c and FEV_1_/FVC ratio) and self-reported statins and blood pressure lowering medication.
