# Supplementary material for: Area-Level Deprivation and Overall and Cause-Specific Mortality: 12 Years’ Observation on British Women and Systematic Review of Prospective Studies
Source: PLoS One. 2013 Sep 24;8(9):e72656. doi: 10.1371/journal.pone.0072656 (PMC3782490; doi:10.1371/journal.pone.0072656)
Supplement: Table S5 — Hazard Ratio (95% CI) of cause-specific death per 1-SD increase in the IMD score among participants with complete information in all co-variables. (DOC) [file pone.0072656.s010.doc]

**Table S5.** Hazard Ratio and 95% Confident Intervals of cause-specific death per 1-SD increase in the IMD score among participants with complete information in all co-variables, N=2,912.

|  | **Model 1** | **Model 2** | **Model 3** | **Model 4** |
| --- | --- | --- | --- | --- |
|
| **Cause of death** | Adjusted  for age | Adjusted for  Model 1 variables plus lifecourse SEP | Adjusted for  Model 2 variables plus  health behaviours^a^ | Adjusted for  Model 3 variables plus biological factors^b^ and CVD medication^c^ |
| **Vascular** | 1.24 (1.09-1.42) | 1.22 (1.05-1.40) | 1.22 (1.04-1.43) | 1.22 (1.03-1.44) |
| **Cancers** | 1.05 (0.92-1.21) | 1.06 (0.91-1.23) | 1.06 (0.90-1.25) | 1.08 (0.92-1.27) |
| **Respiratory** | 1.49 (1.21-1.83) | 1.40 (1.12-1.75) | 1.27 (0.97-1.65) | 1.27 (0.97-1.67) |
| **Other causes** | 1.23 (1.04-1.46) | 1.23 (1.02-1.48) | 1.11 (0.90-1.37) | 1.09 (0.88-1.35) |
| ***All causes*** | 1.20 (1.11-1.30) | 1.19 (1.09-1.29) | 1.15 (1.04-1.26) | 1.15 (1.04-1.26) |

Note: IMD categories were based on the SD from the overall score by country (SD by country were England: 15.7, Wales: 14.3 and Scotland: 16.6

^a^Health-related behaviours included physical activity, alcohol intake, fruit and vegetable intake and concentrations of cotinine.

^b^Biological factors included body mass index , systolic BP, LDL-c and FEV_1_/FVC ratio

^c^CVD medication include self-reported statins and BP lowering medication.

IMD, index of multiple deprivation; SD, standard deviation; SEP, socioeconomic position; BMI, body mass index; BP, blood pressure; LDL-c, low-density lipoprotein cholesterol; FEV_1_/FVC, forced expiratory volume in 1 s/ forced vital capacity ratio; CVD, cardiovascular disease
