# Supplementary material for: Area-Level Deprivation and Overall and Cause-Specific Mortality: 12 Years’ Observation on British Women and Systematic Review of Prospective Studies
Source: PLoS One. 2013 Sep 24;8(9):e72656. doi: 10.1371/journal.pone.0072656 (PMC3782490; doi:10.1371/journal.pone.0072656)
Supplement: Table S6 — Change in the hazard ratio (95% CI) of cause-specific death per 1-SD increase of IMD score after adding waist circumference to the model-4. (DOC) [file pone.0072656.s011.doc]

**Table S6.** Change in the hazard Ratio 95% Confident Intervals of cause-specific death per 1-SD increase of IMD score after adding waist circumference to the model-4

| **Cause of death** | Model-4^a^ alone | Model-4^a^ plus waist circumference |
| --- | --- | --- |
| **Vascular** | 1.22 (1.03-1.44) | 1.23 (1.04-1.45) |
| **Cancers** | 1.08 (0.92-1.27) | 1.08 (0.92-1.27) |
| **Respiratory** | 1.27 (0.97-1.67) | 1.28 (0.98-1.67) |
| **Other causes** | 1.09 (0.88-1.35) | 1.10 (0.89-1.36) |
| ***All causes*** | 1.15 (1.04-1.26) | 1.15 (1.05-1.27) |

^a^Model 4 was adjusted for age, lifecourse socioeconomic position score, physical activity, alcohol intake, fruit and vegetable intake, concentrations of cotinine, body mass index, systolic BP, LDL-c, FEV_1_/FVC ratio and self-reported statins and BP medication.

IMD, index of multiple deprivation; SD, standard deviation; SEP, socioeconomic position; BMI, body mass index; BP, blood pressure; LDL-c, low-density lipoprotein cholesterol; FEV_1_/FVC, forced expiratory volume in 1 s/ forced vital capacity ratio.
