# Supplementary material for: Area-Level Deprivation and Overall and Cause-Specific Mortality: 12 Years’ Observation on British Women and Systematic Review of Prospective Studies
Source: PLoS One. 2013 Sep 24;8(9):e72656. doi: 10.1371/journal.pone.0072656 (PMC3782490; doi:10.1371/journal.pone.0072656)
Supplement: Table S7 — Summary details of published standard prospective studies investigating the association of area-level deprivation and overall or cause-specific mortality. (DOC) [file pone.0072656.s012.doc]

**Table S7**. Summary details of published **standard prospective studies** investigating the association of area-level deprivation and overall or cause-specific mortality

T, tertile; C, categories; D, deciles; Q, quartile or quintile; RR, relative risk; OR, odds ratio; HR, hazard ratio; SD, standard deviation SES, socioeconomic status; NR, not reported

Note: The reference categories are shown underlined. (a) Adjustments were made using social class or risk factor. We only show the data for the measure of area-level deprivation when risk factors were included in the model without social class.

| **First author, year-publication (country)** | **Follow-up (years)** | **No. Individuals** | **Age (range)** | **Measure of area-level deprivation** | **Type of area-level (number)** | **Regression analysis** | **Risk comparison of area-level deprivation** | **Risk ratio (95% CI) minimal adjustment** | **Covariables minimal adjustment** | **Risk ratio (95% CI) maximum adjustment** | **Covariables maximum adjustment (all included age and sex)** |
| --- | --- | --- | --- | --- | --- | --- | --- | --- | --- | --- | --- |
| **Total mortality** | |  |  |  |  |  |  |  |  |  |  |
| Smith et al 1998 (UK) | 15 | 6,961 (men) and 7,991 (women) | 45-64 | Carstairs and Morris index | Postcode sectors (14) | Standard Cox regression | ***C3 (most deprived) vs. C1 (least deprived)*** | Men:  1.47 RR (1.28 -1.68)  Women:  1.40 RR (1.19-1.64) | Age, sex and race | Men:  1.24 RR (1.08-1.42)  Women:  1.16 RR (0.99-1.36) | (a) Diastolic blood pressure, cholesterol, body mass index, FEV1 score, smoking, angina, ECG ischemia, and bronchitis |
| Yen & Kapplan, 1999 (USA) | 11 | 1,129 (men and women) | 36-96 | Neighbourhood social environment score | Neighbourhoods census tract level (NR) | Multilevel logistic regression | ***T1 (low social environment) vs. T2/T3 (high social environment)*** | 1.58 OR (1.15-2.18) | Age and sex | 1.58 OR (1.13-2.24) | Income, education, race/ethnicity, smoking status, body mass index, alcohol consumption, perceived health status |
| Jones et al 2000 (UK) | 12-13 | 3,769 (men) and 4,951 (women) | 16-97 | Customised deprivation index score | Electoral ward (396),constituencies (207) and Regions  ( 22) | Multilevel logistic regression | ***1 unit increase of score (continuous)*** | NR (only maximum adjusted model given) | NR (only maximum adjusted model given) | 1.02 OR (0.023 SE) | Ownership of dwelling, social class, unemployment, smoking, exercise, diet, alcohol |
| Malmstrom et al 2001 (Sweden) | 8 | 22,236 (men and women) | 25-74 | Care Need Index (CNI): Jarman score | Small area markets (8,519) | Standard Cox regression | ***D10 (most deprived) vs. D1 (least deprived)*** | 1.74 RR (1.24-2.45) | Age and sex | 1.19 RR (0.83-1.70) | Socioeconomic position (occupation and working position), housing tenure, marital status, social network |
| Steenland et al, 2004 (USA) | 8 | 179,383 (men and women) | 50-74 | Area-level socioeconomic status score | Census block (NR) | Standard Cox regression | **C1 (low SES) vs.**  **C6 (high SES)** | Men:  1.46 HR (1.32-1.62)  Women:  1.30 HR ( 1.13-1.50) | Age and sex | Men:  1.02 HR (0.91-1.13)  Women:  1.05 HR(0.91-1.21) | Education, race, smoking, marital status, body mass index, exercise, alcohol consumption, fruit/vegetable/saturated fat variables, menopausal status, and hormone use (for women only) |
| Borrell et al, 2004 (USA) | 11-13 | 14,004 (men and women) | 45-64 | Neighbourhood SES composite index | Census block groups (NR) | Standard Cox regression | ***T1 (low SES) vs. T3(high SES)*** | Whites:  1.6 HR (1.3-1.9) African:  1.5 HR (1.2- 1.8) | Age, sex and clinical examination centre | Whites:  1.1 HR (0.9- 1.4)  African:  1.1 HR(0.9- 1.4) | Centre, income, education, occupation. |
| Diez Roux et al, 2004 (USA) | 8 | 5,074 (men and women) | 65+ | Neighbourhood SES composite index | Census block groups (908) | Standard Cox regression | ***T1 (low SES) vs. T3 (high SES)*** | Whites:  1.4 HR (1.2-1.6)  African:  1.4 HR (1.0-2.1) | Age and sex | Whites:  1.2 HR (1.0-1.4)  African:  1.3 HR (0.9-1.9) | Income, education, occupation |
| Morries et al, 2008 (UK) | 18-20 | 5,049 men | 40-59 | Carstairs Deprivation Scores | Electoral Wards (969) | Multilevel Poisson regression | **Q5 (most deprived) vs. Q1 (least deprived)** | 1.64 HR (1.35-2.00) | Age, sex and race | 1.10 HR (0.89-1.34) | Marital status, housing tenure, car ownership, social networks, occupational social class |
| Major et al, 2010 (USA) | 11 | 409,775 (men and women) | 50-71 | Neighbourhood socio-economic deprivation index | Neighbourhoods census tract level ( NR) | Standard Cox regression | **Q5 (most deprived) vs. Q1 (least deprived)** | Men:  1.66 HR (1.58 - 1.75)  Women:  1.53 HR ( 1.43- 1.63) | Age, sex | Men:  1.17 HR (1.10-1.24)  Women:  1.13 HR (1.05-1.22) | Education, race, marital status, BMI, smoking, physical activity, self-reported health status, , energy, alcohol use, fruit, vegetables and meat intakes, vitamin use and menopausal hormone use (for women only) |
| Wight et al, 2010 (USA) | 2 | 3,442 (men and women) | 70+ | Neighbourhood socioeconomic disadvantage index | Census tracts level (1,217) | Multilevel logistic regression | ***1 percentage increase in composite index (continuous)*** | 1.19 OR (1.08-1.31) | Age, sex | 1.14 OR (0.98-1.32) | Marital status, ethnicity, education, household wealth and income. |
| Yao & Robert, 2011 (USA) | 16 | 1,211 ( men and women) | 65+ | Neighbourhood Socioeconomic Disadvantage Index (SDI) | Neighbourhoods census tract level ( NR) | Multilevel Cox regression | ***1 SD increase of SDI score (continuous)*** | 1.18 HR (P< 0.05) | Age, sex and race | 1.09 HR (P< 0.01) | Race, education, family income |
| **Vascular** |  |  |  |  |  |  |  |  |  |  |  |
| Smith et al 1998 (UK) | 15 | 6,961 (men) and 7,991 (women) | 45-64 | Carstairs and Morris index | Postcode sectors (14) | Standard Cox regression | ***C3 (most deprived) vs. C1 (least deprived)*** | Men:  1.33 RR (1.11-1.60)  Women:  1.48 RR (1.17-1.86) | Age, sex and race | Men:  RR=1.19 (0.98,1.43)  Women:  RR=1.21 (0.95,1.52) | Diastolic blood pressure, cholesterol, body mass index, FEV1 score, smoking, angina, ECG ischemia, and bronchitis |
| Steenland et al, 2004 (USA) | 8 | 179,383 (men and women) | 50-74 | Area-level socioeconomic status composite score | Census block (NR) | Standard Cox regression | **C1 (low SES) vs.**  **C6 (high SES)** | Men:  1.86 HR (1.56-2.21)  Women:  1.46 HR (1.10-1.93) | Age, sex | Men:  1.25 HR (1.05- 1.50)  Women:  0.97 HR (0.73-1.29) | Education, race, smoking, marital status, BMI, exercise, alcohol consumption, fruit/vegetable/saturated fat variables, use of aspirin, blood pressure-lowering and cholesterol-lowering medication and menopausal status and hormone use (for women only) |
| Borrell et al, 2004 (USA) | 11-13 | 14,004 (men and women) | 45-64 | Neighbourhood SES composite index | Census block groups (NR) | Standard Cox regression | ***T1 (low SES) vs. T3 (high SES)*** | Whites:  1.9 RR (1.4- 2.7)  African:  1.5 RR (1.0-2.1) | Age, sex and clinical examination centre | Whites:  1.4 RR (1.0- 2.0)  African:  0.9 RR (0.7-1.3) | Centre, income, education, occupation, CVD risk factors and prevalence of CHD at baseline |
| Diez Roux et al, 2004 (USA) | 8 | 5,074 (men and women) | 65+ | Neighbourhood SES composite index | Census block groups (908) | Standard Cox regression | ***T1 (low SES) vs. T3 (high SES)*** | Whites:  1.9 HR (1.5 -2.4)  African:  1.5 HR (0.9 -2.7) | Age and sex | Whites:  1.5 HR (1.2 -1.9)  African:  1.2 HR (0.7- 2.2) | Income, education, occupation |
| Major et al, 2010 (USA) | 11 | 409,775 (men and women) | 50-71 | Neighbourhood socio-economic deprivation index | Neighbourhoods census tract level ( NR) | Standard Cox regression | **Q5 (most deprived) vs. Q1 (least deprived)** | Men:  2.02 HR (1.84-2.23)  Women:  1.84 HR (1.61-2.09) | Age and sex | Men:  1.33 HR (1.19-1.49)  Women:  1.18 HR (1.01-1.38) | Education, race, marital status, BMI, smoking, physical activity, self-reported health status, , energy, cholesterol, alcohol use, fruit, vegetables and meat intakes, vitamin use and menopausal hormone use (for women only) |
|  | **CHD** |  |  |  |  |  |  |  |  |  |  |
| Steenland et al, 2004 (USA) | 8 | 179,383 (men and women) | 50-74 | Area-level socioeconomic status composite score | Census block (NR) | Standard Cox regression | **C1 (low SES) vs.**  **C6 (high SES)** | Men:  1.89 HR (1.51-2.37)  Women:  1.38 HR (0.94-2.03) | Age and sex | Men:  1.25 HR (0.99- 1.58)  Women:  0.87 HR (0.59-1.29) | Education, race, smoking, marital status, body mass index, exercise, alcohol consumption, fruit/vegetable/saturated fat variables, use of aspirin, blood pressure-lowering and cholesterol-lowering medication and menopausal status and hormone use (for women only) |
|  | **Stroke** | |  |  |  |  |  |  |  |  |  |
| Steenland et al, 2004 (USA) | 8 | 179,383 (men and women) | 50-74 | Area-level socioeconomic status composite score | Census block (NR) | Standard Cox regression | **C1 (low SES) vs.**  **C5/C6 (high SES)** | Men:  1.42 HR (1.03-1.97)  Women:  1.19 HR (0.80-1.76) | Age and sex | Men:  1.16 HR (0.82- 1.64)  Women:  0.99 HR (0.66-1.50) | Education, race, smoking, marital status, body mass index, exercise, alcohol consumption, fruit/vegetable/saturated fat variables, use of aspirin, blood pressure-lowering and cholesterol-lowering medication and menopausal status and hormone use (for women only) |
| **Cancer** |  |  |  |  |  |  |  |  |  |  |  |
| Steenland et al, 2004 (USA) | 8 | 179,383 (men and women) | 50-74 | Area-level socioeconomic status composite score | Census block (NR) | Standard Cox regression | **C1 (low SES) vs.**  **C6 (high SES)** | Men:  1.21 HR (1.03-1.42)  Women:  1.14 HR ( 0.93-1.39) | Age and sex | Men:  0.87 HR (0.74-1.03)  Women:  1.02 HR (0.83-1.25) | Education, race, smoking, marital status, body mass index, exercise, alcohol consumption, fruit/vegetable/saturated fat variables, menopausal status, and hormone use (for women only) |
| Borrell et al, 2004 (USA) | 11-13 | 14,004 (men and women) | 45-64 | Neighbourhood SES composite index | Census block groups (NR) | Standard Cox regression | ***T1 (low SES) vs. T3 (high SES)*** | Whites:  1.5 RR (1.2- 2.0)  African:  1.4 RR (1.0-2.1) | Age, sex and clinical examination centre | Whites:  1.1 RR (0.8- 1.5)  African:  1.3 RR (0.9-1.8) | Centre, income, education and occupation |
| Major et al, 2010 (USA) | 11 | 409,775 (men and women) | 50-71 | Neighbourhood socio-economic deprivation index | Neighbourhoods census tract level  ( NR) | Standard Cox regression | **Q5 (most deprived) vs. Q1 (least deprived)** | Men:  1.44 HR (1.32-1.57) Women:  1.27 HR ( 1.15-1.40) | Age and sex | Men:  1.09 HR (1.00-1.20) Women:  1.09 HR (0.99-1.22) | Education, race, marital status, family history of cancer, physical activity, smoking, self-reported health status, BMI, energy, alcohol use, fruit, vegetables and meat intakes, and vitamin use and menopausal hormone use (for women only) |
