# Supplementary material for: Area-Level Deprivation and Overall and Cause-Specific Mortality: 12 Years’ Observation on British Women and Systematic Review of Prospective Studies
Source: PLoS One. 2013 Sep 24;8(9):e72656. doi: 10.1371/journal.pone.0072656 (PMC3782490; doi:10.1371/journal.pone.0072656)
Supplement: Table S8 — Summary details of published record linkage studies investigating the association of area-level deprivation and overall or cause-specific mortality. (DOC) [file pone.0072656.s013.doc]

**Table S8.** Summary details of published **record linkage studies** investigating the association of area-level deprivation and overall or cause-specific mortality

T, tertile; C, categories; D, deciles; Q, quartile or quintile; RR, relative risk; OR, odds ratio; HR, hazard ratio; SD, standard deviation SES, socioeconomic status; NR, not reported

Note: The reference categories are shown underlined

| **First author, year-publication (country)** | **Follow-up (years)** | **No. Individuals** | **Age (range)** | **Measure of area-level deprivation** | **Type of area-level (number)** | **Regression analysis** | **Risk comparison of area-level deprivation** | **Risk ratio (95% CI) minimal adjustment** | **Covariables minimal adjustment** | **Risk ratio (95% CI) maximum adjustment** | **Covariables maximum adjustment (all included age and sex)** |
| --- | --- | --- | --- | --- | --- | --- | --- | --- | --- | --- | --- |
| **Total mortality** | |  |  |  |  |  |  |  |  |  |  |
| Sloggett & Joshi, 1998 (UK) | 11 | More than 300,000 people | 20-64 | Area deprivation score | Electoral ward (5500) | Standard Logistic regression | **1 unit increase in deprivation score** | Men:  1.08 OR (1.06-1.09)  Women:  1.09 OR (1.07-1.11) | Age, sex, time period and north/south zone | Males:  1.02 OR (1.00-1.03) Females:  1.04 OR (1.02-1.06) | Time period, zone, social class (employment in social class 4 or 5), unemployment, car access, home ownership and housewife status |
| Winkleby & Cubbin, 2003 (USA) | 11 | 200,334 (men) and 223,234 (women) | 25-64 | Neighbourhood SES index | Census tract level (NR) | Standard Cox regression | **T1 (low SES) vs. T3 (high SES)** | Black men:  1.52 HR (1.31 -1.77)  White men:  1.59 HR (1.47 -1.72)  Mexican-American men:  1.57 HR (1.16-2.12)  Black women:  1,56 HR (1,30-1,86)  White women:  1.43 HR (1.33-1.55)  Mexican-American women:  1.61 HR (1.11 - 2.34) | Age and sex | Black men:  1.11 HR (0.94 -1.32)  White men:  1.18 HR (1.09-1.27)  Mexican-American men:  1.35 HR (0.96 - 1.90)  Black women:  1.15 HR (0.95-1.38)  White women:  1.11 HR (1.02 - 1.22)  Mexican-American women:  0.95 HR (0.63 - 1.43) | Income, education, and occupational/employment status |
| Curtis et al, 2004 (UK) | 10 | 25,226 (men) and 37,493 (women) | 0-16 in 1939  42-58 in 1981 | Carstairs Deprivation Scores | Electoral ward (192) | Standard Logistic regression | **Q4/Q5 (least affluent) vs. Q1 (most affluent)** | NR (only maximum adjusted model given) | NR (only maximum adjusted model given) | Men:  1.17 OR (1.00-1.36)  Women:  1.36 OR (1.18-1.57) | Social class (occupational classiﬁcations), housing tenure, marital status, unemployed, broad regional location, area unemployment variable in 1930s |
| Marinacci et al, 2004 (Italy) | 10 | 799,564 in 1971-1980; 889,432 1981-1991; 821,736 in 1991 1999 | ≥15 | Neighbourhood deprivation index | Administrative neighbourhoods (23) | Multilevel Poisson regression | **Q4/Q5 (deprived) vs. Q1/Q2 (advantaged)** | Men (15-64 years): 1.20 RR (1.14-1.27)  Men (≥65 years):  1.08 RR (1.04-1.11)  Women (15-64 years):  1.16 RR (1.12-1.19)  Women (≥65 years): 1.08 RR (1.04-1.12) | Age, sex, area of birth and time period | Men (15-64 years): 1.09 RR (1.03 -1.16)  Men (≥65 years):  1.04 RR (1.02-1.07)  Women (15-64 years):  1.08 RR (1.06-1.11)  Women (≥65 years):  1.05 RR (1.01-1.09) | Area of birth, time period, housing conditions and educational level |
| Jaffe et al, 2005 (Israel) | 9.5 | 63,513 (men) and 67,643 (women) | 45-89 | Area-level SES index | Statistical areas (882) | Multilevel logistic regression | **1 unit increase in area SES** | Men (45-69 years): 1.04 OR (1.04–1.05)  Men (70-89 years): 1.02 OR (1.01–1.03)  Women (45-69 years):  1.04 OR (1.03–1.04)  Women (70-89 years): 1.03 OR (1.02–1.04) | Age and sex | Men (45-69 years): 1.02 OR (1.02, 1.03)  Men (70-89 years):  1.01 OR (1.00, 1.02)  Women (45-69 years): 1.02 OR (1.01, 1.02)  Women (70-89 years):  1.02 OR (1.01, 1.03) | Marital status, origin, education, number of rooms in the house, household amenities score |
| Blakely et al, 2006 (New Zealand) | 3 | NR | 25-74 | Neighbourhood deprivation index | Census area units (1,683) and territorial Authorities (73) | Multilevel Poisson regression | **Q5 (Most deprived) vs. Q1 (Least deprived)** | NR (only maximum adjusted model given) | NR (only maximum adjusted model given) | - At neighbourhood level:  Men:  1.45 RR (1.36–1.55)  Women:  1.45 RR (1.34–1.57)  - At regional level:  Men:  1.44 RR (1.36–1.53)  Women:  1.42 RR (1.31–1.53) | Marital status, ethnicity, income, education, car access, labour force, rurality, neighbourhood volunteerism |
| Turrell et al, 2007, (Australia) | 4 | 3,393,237 (men) and 2,602,424 (women) | 25–64 | Index of Relative Socio-economic Disadvantage (IRSD) | statistical local areas (1,317) | Multilevel binomial logit-link | **Q5 (Most disadvantaged) vs. Q1 (Least disadvantaged)** | Men:  1.91 RR (1.78-2.04)  Women:  1.53 RR (1.39-1.68) | Age and sex | Men:  1.59 RR (1.48-1.70)  Women:  1.48 RR (1.35-1.63) | Occupation |
| **Vascular** |  |  |  |  |  |  |  |  |  |  |  |
|  | **CHD** |  |  |  |  |  |  |  |  |  |  |
| Marinacci et al, 2004 (Italy) | 10 | 799,564 in 1971-1980; 889,432 1981-1991; 821,736 in 1991 1999 | ≥15 | Neighbourhood deprivation index | Administrative neighbourhoods (23) | Multilevel Poisson regression | **Q4/Q5 (deprived) vs. Q1/Q2 (advantaged)** | Men (15-64 years): 1.15 RR (1.09-1.21)  Men (≥65 years):  1.00 RR (0.95-1.06)  Women (15-64 years):  1.27 RR (1.13-1.43)  Women (≥65 years): 1.11 RR (1.00-1.22) | Age, sex, area of birth and time period | Men (15-64 years): 1.09 RR (1.02-1.16)  Men (≥65 years):  1.00 RR (0.95-1.04)  Women (15-64 years):  1.15 RR (1.03-1.28)  Women (≥65 years):  1.09 RR (0.98-1.21) | Area of birth, time period, housing conditions and educational level |
| Petrelli et al., 2006 (Italy) | 6 | 250,692 (men) and 273,063 (women) | 35–74 | Neighbourhood deprivation index | Administrative neighbourhoods (23) | Hierarchical Poisson regression | **Q4/Q5 (Most deprived) vs. Q1/Q2 (Least deprived)** | NR (only maximum adjusted model given) | NR (only maximum adjusted model given) | Men:  1.14 RR (0.96-1.36)  Women:  1.04 RR (0.86-1.25) | Area of birth, educational level, job status and income |
| **Respiratory** |  |  |  |  |  |  |  |  |  |  |  |
| Marinacci et al, 2004 (Italy) | 10 | 799,564 in 1971-1980; 889,432 1981-1991; 821,736 in 1991 1999 | ≥15 | Neighbourhood deprivation index | Administrative neighbourhoods (23) | Multilevel Poisson regression | **Q4/Q5 (deprived) vs. Q1/Q2 (advantaged)** | Men (15-64 years): 1.29 RR (1.14-1.47)  Men (≥65 years):  1.12 RR (0.94-1.33)  Women (15-64 years):  1.45 RR (1.21-1.73)  Women (≥65 years): 1.09 RR (1.01-1.17) | Age, sex, area of birth and time period | Men (15-64 years): 1.20 RR (1.07-1.35)  Men (≥65 years):  1.04 RR (0.87-1.24)  Women (15-64 years):  1.24 RR (1.04-1.48)  Women (≥65 years):  1.03 RR (0.96-1.12) | Area of birth, time period, housing conditions and educational level |
| **Cancer** |  |  |  |  |  |  |  |  |  |  |  |
| Bentley et al, 2008, (Australia) | 4 | 3,393,237 (men) and 2,602,424 (women) | 25–64 | Index of Relative Socio-economic Disadvantage (IRSD) | Statistical Local Areas (1,317) | Multilevel binomial logit-link | **Q5 (Most disadvantaged) vs. Q1 (Least disadvantaged)** | Men:  1.69 RR (1.54-1.84)  Women: 1.31 RR (1.19-1.44) | Age and sex | Men:  1.48 (1.35-1.63)  Women:  1.30 RR (1.18-1.43) | Occupation, State and Territory |
