# Supplementary material for: Area-Level Deprivation and Overall and Cause-Specific Mortality: 12 Years’ Observation on British Women and Systematic Review of Prospective Studies
Source: PLoS One. 2013 Sep 24;8(9):e72656. doi: 10.1371/journal.pone.0072656 (PMC3782490; doi:10.1371/journal.pone.0072656)
Supplement: Table S9 — Reporting specific-causes of mortality from studies included in the systematic review. (DOC) [file pone.0072656.s014.doc]

**Table S9.** Reporting specific-causes of mortality from studies included in the systematic review

| **First author** | **All causes** | **Vascular** | | **Cancer** | **Respiratory** |
| --- | --- | --- | --- | --- | --- |
|  |  | **CHD** | **Stroke** |  |  |
| **Standard Prospective** | | | | | |
| Smith et al. | **√** | **√** | | - | - |
| Yen et al. | **√** | - | - | - | - |
| Jones et al. | **√** | - | - | - | - |
| Malmstrom et al. | **√** | - | - | - | - |
| Steenland et al.^a^ | **√** | **√** | **√** | **√** | - |
| Borrell et al. | **√** | **√** | | **√** | - |
| Diez-Roux et al. | **√** | **√** | | - | - |
| Morris et al. | **√** | - | - | - | - |
| Major et al. | **√** | **√** | | **√** | - |
| Wight et al. | **√** | - | - | - | - |
| Yao et al. | **√** | - | - | - | - |
| **Record Linkage** | | | | | |
| Sloggett et al. | **√** | - | - | - | - |
| Winkleby et al. | **√** | - | - | - | - |
| Curtis et al. | **√** | - | - | - | - |
| Marinacci et al. | **√** | **√** | - | - | **√** |
| Jaffe et al. | **√** | - | - | - | - |
| Blakely et al. | **√** | - | - | - | - |
| Petrelli et al. | - | **√** | - | - | - |
| Turrell et al. | **√** | - | - | - | - |
| Bentley et al. | - | - | - | **√** | - |

^a^Vascular mortality data were also available.

Studies classified as standard prospective are in webreferences 2, 3, 4, 5, 11, 7, 9, 17, 18, 19 and 20 and those as record linkage in webreferences 1, 6, 8, 10, 12, 13, 14, 15 and 16 in **Text S1**.

CHD, coronary heart disease
