# Supplementary material for: Area-Level Deprivation and Overall and Cause-Specific Mortality: 12 Years’ Observation on British Women and Systematic Review of Prospective Studies
Source: PLoS One. 2013 Sep 24;8(9):e72656. doi: 10.1371/journal.pone.0072656 (PMC3782490; doi:10.1371/journal.pone.0072656)
Supplement: Table S10 — Reporting individual level socioeconomic position and area-level confounder from studies included in the systematic review. (DOC) [file pone.0072656.s015.doc]

**Table S10.** Reporting individual level socioeconomic position and area-level confounder from studies included in the systematic review

| **First author** | **Adulthood SEP** | | | |  | **Area-level confounder** | | |
| --- | --- | --- | --- | --- | --- | --- | --- | --- |
|  | **E** | **I** | **O** | **Others** |  | **Reporting** | **Description** | |
| **Standard Prospective** |  |  |  |  |  |  |  | |
| Smith et al. | - | - | **√** | - |  | - | - | |
| Yen et al. | **√** | **√** | - | - |  | - | - | |
| Jones et al. | - | - | **√** | Ownership of dwelling and unemployment |  | - | - | |
| Malmstrom et al. | - | - | **√** | Housing tenure |  | - | - | |
| Steenland et al. | **√** | - | - | - |  | - | - | |
| Borrell et al. | **√** | **√** | **√** | - |  | **√** | Clinical examination centre | |
| Diez-Roux et al. | **√** | **√** | **√** | - |  | - | - | |
| Morris et al. | - | - | **√** | Car ownership and housing tenure |  | - | - | |
| Major et al. | **√** | - | - | - |  | - | - | |
| Wight et al. | **√** | **√** | - | Household wealth |  | - | - | |
| Yao et al. | **√** | **√** | - | - |  | - | - | |
| **Record Linkage** |  |  |  |  |  |  |  |  |
| Sloggett et al. | - | - | **√** | Unemployment, car access, home ownership and housewife status |  | - | - | |
| Winkleby et al. | **√** | **√** | **√** | - |  | - | - | |
| Curtis et al. | - | - | **√** | Housing tenure and unemployment |  | **√** | Broad regional location and area unemployment variable in childhood | |
| Marinacci et al. | **√** | - | - | Housing conditions |  | - | - | |
| Jaffe et al. | **√** | - | - | Rooms in the house and household amenities |  | - | - | |
| Blakely et al. | **√** | **√** | - | Car access, labour force |  | **√** | Rurality and neighbourhood volunteerism | |
| Petrelli et al. | **√** | **√** | **√** | - |  | **√** | Area of birth | |
| Turrell et al. | - | - | **√** | - |  | - | - | |
| Bentley et al. | - | - | **√** | - |  | **√** | State and Territory level | |

SEP, Socioeconomic position; E, education; I, income; O, occupation

Studies classified as standard prospective are in webreferences 2, 3, 4, 5, 11, 7, 9, 17, 18, 19 and 20 and those as record linkage in webreferences 1, 6, 8, 10, 12, 13, 14, 15 and 16 in **Text S1**.
