# Supplementary material for: Area-Level Deprivation and Overall and Cause-Specific Mortality: 12 Years’ Observation on British Women and Systematic Review of Prospective Studies
Source: PLoS One. 2013 Sep 24;8(9):e72656. doi: 10.1371/journal.pone.0072656 (PMC3782490; doi:10.1371/journal.pone.0072656)
Supplement: Table S11 — Summary relative risks (95% CI) for area-level deprivation and specific health outcomes derived from standard prospective and record-linkage studies. (DOC) [file pone.0072656.s016.doc]

**Table S11**. Summary relative risks (95% CI) for area-level deprivation and specific health outcomes derived from standard prospective and record-linkage studies. These results include data from the BWHHS.

| **Outcomes** | **Standard prospective studies** | | |  | **Record-linkage studies** | | |  | **Overall** | | |
| --- | --- | --- | --- | --- | --- | --- | --- | --- | --- | --- | --- |
|  | **No. of Studies** | **Summary RR**  **(95%CI)** | **I^2^** |  | **No. of Studies** | **Summary RR**  **(95%CI)** | **I^2^** |  | **No. of Studies** | **Summary RR**  **(95%CI)** | **I^2^** |
| **All causes Mortality** |  |  |  |  |  |  |  |  |  |  |  |
| Minimal Adjustment | 11 | 1.17  (1.13-1.21) | 70.2%,  p= 0.000 |  | 5 | 1.09  (1.05-1.14) | 93.2 %,  p=0.000 |  | 16 | 1.15  (1.11-1.19) | 93.2 %  p= 0.000 |
| Maximal Adjustment | 12 | 1.06  (1.03-1.08) | 43.9 %,  p= 0.051 |  | 7 | 1.07  (1.03-1.10) | 94.6 %  p=0.000 |  | 19 | 1.06  (1.04.1.08) | 86.7 %  p=0.000 |
| **Vascular** |  |  |  |  |  |  |  |  |  |  |  |
| Minimal Adjustment | 6 | 1.24  (1.20-1.28) | 26.6%  p=0.235 |  | 1 | 1.04  (1.01-1.08) | N/A |  | 7 | 1.21  (1.12-1.31) | 92.5%  p=0.000 |
| Maximal Adjustment | 6 | 1.12  (1.07-1.18) | 47.6%  p=0.126 |  | 2 | 1.02  (1.00-1.05) | 0.0%  p=0.361 |  | 8 | 1.09  (1.04-1.14) | 80.3.%  p=0.000 |
| **Cancer** |  |  |  |  |  |  |  |  |  |  |  |
| Minimal Adjustment | 4 | 1.11  (1.07-1.15) | 88.95%  p=0.321 |  | 1 | 1.14  (1.05-1.24) | N/A |  | 5 | 1.11  (1.08-1.14) | 0.0%  p=0.417 |
| Maximal Adjustment | 4 | 1.02  (0.99-1.06) | 29.2%  p=0.237 |  | 1 | 1.12  (1.07-1.17) | N/A |  | 5 | 1.05  (1.00-1.11) | 75.7%  p=0.002 |
| **Respiratory** |  |  |  |  |  |  |  |  |  |  |  |
| Minimal Adjustment | 1 | 1.51  (1.29-1.74) | N/A |  | 1 | 1.07  (1.02-1.11) | N/A |  | 2 | 1.29  (0.90-1.77) | 94.7%  p=0.000 |
| Maximal Adjustment | 1 | 1.25  (1.97-1.63) | N/A |  | 1 | 1.04  (1.01-1.07) | N/A |  | 2 | 1.09  (0.93-1.27) | 47.5%  p=0.167 |

Studies classified as standard prospective are in webreferences 2, 3, 4, 5, 7, 9, 11, 17, 18, 19 and 20 and those as record linkage in webreferences 1, 6, 8, 10, 12, 13, 14, 15 and 16 in **Text S1**

BWHHS, British Women Health and Heart Study; N/A: not applicable
