# Supplementary material for: Area-Level Deprivation and Overall and Cause-Specific Mortality: 12 Years’ Observation on British Women and Systematic Review of Prospective Studies
Source: PLoS One. 2013 Sep 24;8(9):e72656. doi: 10.1371/journal.pone.0072656 (PMC3782490; doi:10.1371/journal.pone.0072656)
Supplement: Text S1 — Supporting Methods; Supporting Results; Supporting References. (DOC) [file pone.0072656.s017.doc]

**Text S1. Systematic review and meta-analysis of area-level deprivation and cause-specific mortality**

**Methods**

**Search strategy in PubMed:**

**#1 search** ("neighborhood"[Title/Abstract] OR "neighbourhood"[Title/Abstract] OR "area-level"[Title/Abstract])

**#2 search** ("mortality"[Title/Abstract] OR "death"[Title/Abstract])

**#3 search** #1 AND #2

**#4 limit** #3 to English language

**Study Selection**

Citations identified were reviewed using the abstract or full text as necessary by two investigators (MTSS, MMF) to decide its inclusion in the meta-analysis. Only human studies written in English were included. Inclusion criteria of studies were: (1) original article (commentaries, reviews, and editorials were excluded) (2) prospective study design (standard prospective or record linkage); (3) using an area-level deprivation composite index variable (i.e. that combine the multidimensional nature of area-level socioeconomic position into a single measure) (4) controlled for at least one variable at the individual level and (5) reporting data on at least one of the following outcomes: all cause of death, vascular (all-vascular, stroke or coronary heart disease (CHD)), all-cancers or respiratory mortality. We excluded studies focusing only on children and non-general population. Disagreements about inclusion of studies were resolved by consulting a third author (JPC).

**Data extraction**

Information from the indentified studies was entered into a single dataset by two authors (MTSS and MMF) including: 1) author, publication year and country, 2) length of follow-up in years, 3) number of participants, 4) age of the participants, 5) measure of area-level deprivation, 6) type of area-level (geographical units) used in the analysis, 7) type of regression model, 8) type of risk comparison, 9) risk ratios in minimal and maximal adjustment models, 10) degree of adjustment and variables adjusted for. Disagreements about data extracted were resolved by consulting a third author (JPC). When data was not clearly reported, we contacted authors to obtain the information. Only one author was contacted and replied with the relevant data (web-reference [10])

**Statistical analysis**

We conducted a meta-analysis of studies meeting our inclusion criteria alongside the BWHHS results. The majority of the studies provided estimates in relative risks (RR), odds ratios (OR) and hazard ratios (HRs). Estimates from the published studies were presented in various risk comparison types, including the contrast of extreme quartiles, tertiles, lowest versus highest categories, continuous and binary groups. We assumed a log-linear relationship between area-level deprivation and mortality (as observed in the BWHHS) and converted each published estimate into a homogenous risk comparison according to a method described by Danesh et al 1998 [1]. Based on this, we generated for each study a RR for the top-tertile (least deprived, or alternatively most affluent – reference group) versus the bottom tertile (most deprived, or alternatively least affluent –exposure group). Studies were made comparable by taking the inverse of the estimates in some studies and converting all findings into a common direction, where the higher the score represents the least deprived (most affluent alternatively), and the lower score represents the most deprived (least affluent). If studies provided more than one RR (e.g. by gender), these were pooled into a single estimate within each study. From each publication, we extracted the RR from a minimal adjusted model (age and sex and race for multiethnic studies), as well as from the most adjusted model for each of the following outcomes: total mortality, vascular deaths, cancer deaths and respiratory deaths. Variables adjusted for in each model from each study were retrieved and categorized accordingly.

Summary RR and 95% confidence interval (CI) were calculated using the random-effect models. The degree of heterogeneity was examined using I^2^ statistics and small study bias was assessed by a contour enhanced meta-analysis funnel plot, Begg’s regression test and the “Trim-and-Fill” simulation method [2,3] to account for missing studies. Study features such as type-of area-level measure of deprivation, geographical units, study size in the number of cases reported, and type of study (standard prospective or record-linkage) were used for sub-group analysis to explore sources of statistical heterogeneity. The Stata (StataCorp LP) command “metareg” [4] was used for sub-group analysis.

**Results**

**Selection of studies**

A total of 768 articles were identified by using the search strategy. By screening title and abstract, we excluded 726 which did not meet inclusion criteria. Out of 42 full-text articles assessed (18 standard prospective studies and 24 record linkage studies), 20 articles were included in the systematic review. Most common reason for exclusion of studies was the use of a single indicator as measure of area-level deprivation, such as: income, education or occupation. Among those excluded, two studies focused on non general population (colorectal cancer cases), two had irrelevant outcome (cervical cancer mortality and case-fatality from CHD after incidence), two had inappropriate design and two studies had overlapping study populations. See **Figure S2** for details of the selection process and reasons for exclusion.

**Study characteristics**

Main characteristics of selected studies are reported in **Tables S7** and S**8**. A total 18 published studies reported overall mortality, seven reported vascular mortality, four reported all-cancers and one reported respiratory disease mortality. No single study reported all major causes of mortality (**Table S9**). No single study used childhood socioeconomic position (SEP), and degree of adjustment for individual deprivation in middle-aged/elderly adults is reported in **Table S10**. Only five studies adjusted for area-level confounders (**Table S10**). None of the prospective record-linkage studies adjusted for health-related behaviours, while six of twelve standard prospective studies adjusted for at least one behavioural factor and only 2 adjusted for all the four major health-related behaviours (physical activity, alcohol consumption, smoking and fruit and vegetable consumption) (**Tables S7 and S8**).

**Meta-analysis**

Summary RR for overall mortality from studies reporting minimal adjustments, was 1.15 (95% CI: 1.11-1.19), and it was 1.06 (95% CI: 1.04-1.08) for studies reporting maximal adjustments. Heterogeneity with I^2^ statistics was 93.2 % for studies reporting minimal adjustments and 70.2% for studies reporting maximal adjustments (**Figures S3 and S4**). The Begg’s regression test suggested no evidence of publication bias (p=0.310). The “trim-and-fill” simulation method added 8 missing studies to assume symmetry in the funnel plots and suggested the effect estimates with maximum adjustments would have been 1.06 (95% CI: 1.04 -1.08) in a random effect model. This estimated value is close to the value obtained from studies with more than 10,000 cases (1.06 (95% CI: 1.03-1.09); **Figure 3** reported in main manuscript), indicating that this value is unlikely to be influenced by small study bias.

Summary RR with maximal adjustment was 1.09 (95% CI 1.04-1.14) for vascular mortality, 1.05 (95 % CI: 1.00-1.11) for cancer mortality and 1.09 (95% CI: 0.93-1.27) for respiratory mortality. Results were also stratified by level of adjustment and type of prospective studies for each outcome (**Table S11**).

**References of studies included in the Data analysis section**

1. Danesh J, Collins R, Appleby P, Peto R (1998) Association of fibrinogen, C-reactive protein, albumin, or leukocyte count with coronary heart disease: meta-analyses of prospective studies. JAMA 279: 1477-1482.

2. Duval S, Tweedie R (2000) Trim and fill: A simple funnel-plot-based method of testing and adjusting for publication bias in meta-analysis. Biometrics 56: 455-463.

3. Duval S, Tweedie R (2000) A Nonparametric “Trim and Fill” Method of Accounting for Publication Bias in Meta-Analysis. Journal of the American Statistical Association 95: 89-98.

4. Harbord RM, Higgins JPT (2008) Meta-regression in Stata. Stata Journal 8: 493-519.

**Web-references of studies included in the systematic review**

1. Sloggett A, Joshi H. Deprivation indicators as predictors of life events 1981-1992 based on the UK ONS Longitudinal Study. J Epidemiol Community Health 1998;52: 228-33.

2. Smith GD, Hart C, Watt G, Hole D, Hawthorne V. Individual social class, area-based deprivation, cardiovascular disease risk factors, and mortality: the Renfrew and Paisley Study. J Epidemiol Community Health 1998;52: 399-405.

3. Yen IH, Kaplan GA. Neighborhood social environment and risk of death: multilevel evidence from the Alameda County Study. Am J Epidemiol 1999;149: 898-907.

4. Jones K, Gould MI, Duncan C. Death and deprivation: an exploratory analysis of deaths in the health and lifestyle survey. Soc Sci Med 2000;50: 1059-79.

5. Malmstrom M, Johansson SE, Sundquist J. A hierarchical analysis of long-term illness and mortality in socially deprived areas. Soc Sci Med 2001;53: 265-75.

6. Winkleby MA, Cubbin C. Influence of individual and neighbourhood socioeconomic status on mortality among black, Mexican-American, and white women and men in the United States. J Epidemiol Community Health 2003;57: 444-52.

7. Borrell LN, Diez Roux AV, Rose K, Catellier D, Clark BL. Neighbourhood characteristics and mortality in the Atherosclerosis Risk in Communities Study. Int J Epidemiol 2004;33: 398-407.

8. Curtis S, Southall H, Congdon P, Dodgeon B. Area effects on health variation over the life-course: analysis of the longitudinal study sample in England using new data on area of residence in childhood. Soc Sci Med 2004;58: 57-74.

9. Diez Roux AV, Borrell LN, Haan M, Jackson SA, Schultz R. Neighbourhood environments and mortality in an elderly cohort: results from the cardiovascular health study. J Epidemiol Community Health 2004;58: 917-23.

10. Marinacci C, Spadea T, Biggeri A, Demaria M, Caiazzo A, Costa G. The role of individual and contextual socioeconomic circumstances on mortality: analysis of time variations in a city of north west Italy. J Epidemiol Community Health 2004;58: 199-207.

11. Steenland K, Henley J, Calle E, Thun M. Individual- and area-level socioeconomic status variables as predictors of mortality in a cohort of 179,383 persons. Am J Epidemiol 2004;159: 1047-56.

12. Jaffe DH, Eisenbach Z, Neumark YD, Manor O. Individual, household and neighborhood socioeconomic status and mortality: a study of absolute and relative deprivation. Soc Sci Med 2005;60: 989-97.

13. Blakely T, Atkinson J, Ivory V, Collings S, Wilton J, Howden-Chapman P. No association of neighbourhood volunteerism with mortality in New Zealand: a national multilevel cohort study. Int J Epidemiol 2006;35: 981-9.

14. Petrelli A, Gnavi R, Marinacci C, Costa G. Socioeconomic inequalities in coronary heart disease in Italy: a multilevel population-based study. Soc Sci Med 2006;63: 446-56.

15. Turrell G, Kavanagh A, Draper G, Subramanian SV. Do places affect the probability of death in Australia? A multilevel study of area-level disadvantage, individual-level socioeconomic position and all-cause mortality, 1998-2000. J Epidemiol Community Health 2007;61: 13-9.

16. Bentley R, Kavanagh AM, Subramanian SV, Turrell G. Area disadvantage, individual socio-economic position, and premature cancer mortality in Australia 1998 to 2000: a multilevel analysis. Cancer Causes Control 2008;19: 183-93.

17. Morris RW, Wannamethee G, Lennon LT, Thomas MC, Whincup PH. Do socioeconomic characteristics of neighbourhood of residence independently influence incidence of coronary heart disease and all-cause mortality in older British men? Eur J Cardiovasc Prev Rehabil 2008;15: 19-25.

18. Major JM, Doubeni CA, Freedman ND, et al. Neighborhood socioeconomic deprivation and mortality: NIH-AARP diet and health study. PLoS One 2010;5: e15538.

19. Wight RG, Cummings JR, Karlamangla AS, Aneshensel CS. Urban neighborhood context and mortality in late life. J Aging Health 2010;22: 197-218.

20. Yao L, Robert SA. Examining the Racial Crossover in Mortality between African American and White Older Adults: A Multilevel Survival Analysis of Race, Individual Socioeconomic Status, and Neighborhood Socioeconomic Context. J Aging Res 2011;2011: 132073.
